# Analgesic Alkaloids Derived From Traditional Chinese Medicine in Pain Management

**DOI:** 10.3389/fphar.2022.851508

**Published:** 2022-05-10

**Authors:** Wei Jiang, Mingze Tang, Limin Yang, Xu Zhao, Jun Gao, Yue Jiao, Tao Li, Cai Tie, Tianle Gao, Yanxing Han, Jian-Dong Jiang

**Affiliations:** ^1^ Zhejiang Zhenyuan Pharmaceutical Co., Ltd., Shaoxing, China; ^2^ State Key Laboratory of Bioactive Substances and Function of Natural Medicine, Institute of Materia Medica, Chinese Academy of Medical Sciences, Beijing, China; ^3^ First Clinical Division, Peking University Hospital of Stomatology, Beijing, China; ^4^ Department of Neurosurgery, Peking Union Medical College Hospital, Chinese Academy of Medicine Sciences & Peking Union Medical College, Beijing, China; ^5^ Beijing Key Laboratory of Traditional Chinese Medicine Basic Research on Prevention and Treatment of Major Diseases, Experimental Research Center, China Academy of Chinese Medical Sciences, Beijing, China; ^6^ State Key Laboratory of Coal Resources and Safety Mining, China University of Mining and Technology, Beijing, China; ^7^ School of Chemical and Environmental Engineering, China University of Mining and Technology, Beijing, China; ^8^ Research Unit of Digestive Tract Microecosystem Pharmacology and Toxicology, Chinese Academy of Medical Sciences, Beijing, China

**Keywords:** analgesics, TCM (trad. Chinese medicine), chronic pain, combinational therapy, alkaloids

## Abstract

Chronic pain is one of the most prevalent health problems. The establishment of chronic pain is complex. Current medication for chronic pain mainly dependent on anticonvulsants, tricyclic antidepressants and opioidergic drugs. However, they have limited therapeutic efficacy, and some even with severe side effects. We turned our interest into alkaloids separated from traditional Chinese medicine (TCM), that usually act on multiple drug targets. In this article, we introduced the best-studied analgesic alkaloids derived from TCM, including tetrahydropalmatine, aloperine, oxysophocarpine, matrine, sinomenine, ligustrazine, evodiamine, brucine, tetrandrine, Stopholidine, and lappaconitine, focusing on their mechanisms and potential clinical applications. To better describe the mechanism of these alkaloids, we adopted the concept of drug-cloud (dCloud) theory. dCloud illustrated the full therapeutic spectrum of multitarget analgesics with two dimensions, which are “direct efficacy”, including inhibition of ion channels, activating γ-Aminobutyric Acid/opioid receptors, to suppress pain signal directly; and “background efficacy”, including reducing neuronal inflammation/oxidative stress, inhibition of glial cell activation, restoring the balance between excitatory and inhibitory neurotransmission, to cure the root causes of chronic pain. Empirical evidence showed drug combination is beneficial to 30–50% chronic pain patients. To promote the discovery of effective analgesic combinations, we introduced an ancient Chinese therapeutic regimen that combines herbal drugs with “Jun”, “Chen”, “Zuo”, and “Shi” properties. In dCloud, “Jun” drug acts directly on the major symptom of the disease; “Chen” drug generates major background effects; “Zuo” drug has salutary and supportive functions; and “Shi” drug facilitates drug delivery to the targeted tissue. Subsequently, using this concept, we interpreted the therapeutic effect of established analgesic compositions containing TCM derived analgesic alkaloids, which may contribute to the establishment of an alternative drug discovery model.

## 1 Introduction

### 1.1 Background

#### 1.1.1 Social Impact of Chronic Pain

Chronic pain refers to pain that recurs over 3 months, which includes scenarios such as neuropathic pain, fibromyalgia, cancer pain and chronic arthritis pain (Treede et al., 2019). Anyone can suffer from chronic pain due to illness, trauma or surgery. Unfortunately, regardless of the substantial medical progress has been made in recent decades, management of chronic pain remained to be one of the most challenging clinical demands. In China, chronic pain affects around 400 million widely distributed patients (Zheng et al., 2020). However, less than 60% of them sought for medical treatment and of only 20% of the patients who received therapy reached sufficient pain relief (Chen et al., 2016; Zheng et al., 2020).

At an individual level, chronic pain limits the mobility and restricts patients’ essential daily activities such as eating, sleeping, and exercising, rendering many of them losing working abilities. In long term, patients’ quality of life is reduced, with impairment in their mental health (Mills et al., 2019). For instance, from 28.6 to 70% of fibromyalgia patients suffering from major depression, which is much higher than that of healthy population (5%) (Alciati et al., 2012; Gracely et al., 2012). At a society level, chronic pain imposed a huge challenge to the public healthcare system. A study in 2021 showed that in the United States, pain induced productivity loss was estimated to be $296 billion per year, which is greater than cardiovascular disease, cancer, or diabetes (Yong et al., 2021).

### 1.2 Pain Spots in Pain Medicine

#### 1.2.1 Current Medication Is Unsatisfactory

Currently, treatments for chronic pain are dependent on anticonvulsants, tricyclic antidepressants, and opioidergic drugs.

Anticonvulsants (such as gabapentin and pregabalin) are the first-line choice for chronic pain management. However, their therapeutic efficacy is unsatisfactory: for traumatic nerve injury, pain was moderately relieved in less than 50% of the patients (Gordh et al., 2008); for mixed nerve pain, significant pain relief was achieved in 21% of patients (Serpell, 2002); for banded neuralgia, effectiveness was recorded in 32–34% of patients (Rice and Maton, 2001). In addition, incidences of neurological and systemic adverse reactions of gabapentin and pregabalin have been increasingly reported in recent years, and these adverse reactions were positively correlated with drug dosages (Chen and Chen, 2014).

Tricyclic antidepressants (TCAs), such as amitriptyline and nortriptyline, block the reuptake of norepinephrine (NA) and serotonin (5-HT) in nerve endings, to increase the available amount of inter-synaptic NA and 5-HT in key brain areas and thus modulate pain signals (Bair et al., 2003; Tamano et al., 2016). TCAs have been widely used as off-label drugs for many chronic pain conditions, although its exact analgesic mechanism (which may involve descending noradrenergic inhibitory pathway) remained to be largely unclear (Bair et al., 2003; Obata, 2017). In most studies, TCAs exhibit only limited potency with modest symptom improvements (Moore et al., 2012). Additionally, their usage is often restricted by bothersome anticholinergic side effects (including sedation, drowsiness, dry mouth, and constipation) (Till et al., 2019). Thus, in practice, TCAs are not universally applicable, but favoring a portion of patients who have comorbid psychological conditions (Till et al., 2019).

On the other hand, opioids can induce strong analgesic effects. But as a trade-off, they have crucial side effects, including tolerance, dependence, and addiction, limiting their usage as a long-term solution (Birnbaum et al., 2011; Vowles et al., 2015; Mercadante et al., 2019). Furthermore, inadequate restrictions on prescriptions of opioids (mainly as pain killer) in recent years, has led to an ongoing opioid crisis especially in western countries (Birnbaum et al., 2011). In the United States, drug overdoses resulted in 70,237 deaths during 2017 (surpassed the number of deaths caused by gun crimes or car accidents); among these, 47,600 (67.8%) involved opioids, with an increasing rate of 12.0% (from 2016 to 2017) (Scholl et al., 2018).

#### 1.2.2 Failed Drug Discovery Model

There are currently about 3,500 clinical trials with chronic pain as indication has been registered worldwide (2021, https://clinicaltrials.gov). However, the path to new drug registration was not smooth. During 2001 to 2009, only few analgesics were approved by the United States Food and Drug Administration (FDA), which are mainly new formulations of existing analgesics (such as slow-release version of opioids) (Kinch, 2015). In 2018, FDA approved a total of 59 new drugs and therapies (highest numbers that breaking all-time records), with three pain killers approved for migraine (Kinch and Griesenauer, 2019). In 2019, FDA approved 48 novel drugs, with two pain drugs approved for migraine (Ebied et al., 2020). In 2020, FDA approved 53 novel therapeutics (the second highest total ever), with two pain drugs approved for migraine, and one for acute pain (Mullard, 2021). To some extent, the lack of “First in Class” or “Me-Only” pain killers manifested the frustration of current drug discovery model (Csermely et al., 2005; Kinch and Griesenauer, 2019; Ebied et al., 2020; Mullard, 2021).

In the current model, as a standard route, scientists first identify genes associated with the disease through literature search, and determine the biological target based on one of the relevant genes, then use the computer-assisted tools to design molecules that could interact with the target, and finally perform preclinical and clinical studies to validate if the compound could meet the requirements for new drug registration (Csermely et al., 2005).

### 1.3 Deep Reasoning of Conundrum

#### 1.3.1 Chronic Pain Mechanism Is Complex

Resembling cancer/metabolic diseases, chronic pain also has multi-facet major symptoms with complicated root causes. So far, the pathology of chronic pain is still not entirely clear, but mechanisms of inflammation, oxidative stress, glial cell activation, and dysregulation of neurotransmitters are involved (Ji et al., 2016; Yang and Chang, 2019; Kaushik et al., 2020).

**FIGURE 1:**
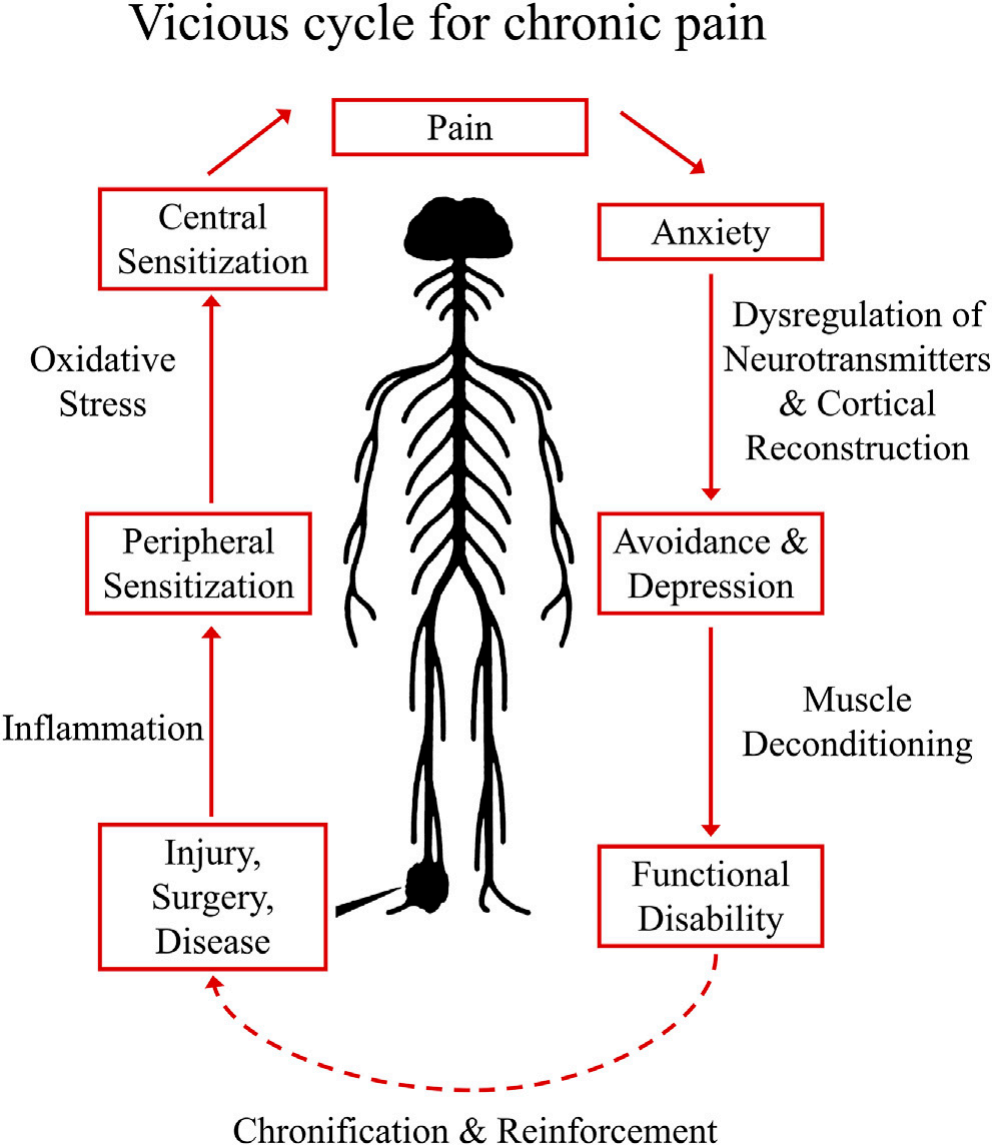
Vicious Cycle for Chronic Pain Establishment. The establishment of chronic pain usually starts with injury, surgery, or diseases that could harm the sensory circuit of the nervous system. When these situations were left to be treated, concomitant local inflammation will sensitize peripheral nociceptors (peripheral sensitization), and with accumulated oxidative stress in the CNS, central sensitization (sensitized pain pathway in the spinal cord) may occur, enhancing the pain perception to an intolerable degree (eventually pain symptoms become a disease). In cognitive part, sustained pain could generate anxiety, the pathological sign of chronic pain at this stage may involve dysregulation of neurotransmitters in the CNS and cortical reconstruction, subsequently result in depression and avoidance behaviors. In long term, symptoms of muscle spam and deconditioning could also occur, causing functional disability. In this vicious cycle, the pain syndrome eventually undergoing a chronification/reinforcement process and becoming increasingly robust.

The development and maintenance of chronic pain is illustrated in a self-reinforcing vicious cycle (Figure 1), in which acute pain eventually undergoing a chronification process and become increasingly robust.

Establishment of chronic pain usually starts with injury, surgery, inflammation, or diseases that harm the sensory circuit of the nervous system. These insults induce the release of neurotransmitters, lipid mediators, fragments of the complement system, neurotrophic factors, cytokines and chemokines (Silva et al., 2017). When these situations are not well managed, local inflammation will sensitize peripheral nociceptors causing peripheral sensitization (Silva et al., 2017). Sensitized primary afferents are responsible for releasing neurotransmitters (including glutamate, substance P, calcitonin gene–related peptide, and brain-derived growth factor) that gradually produce a state of neuronal hyperactivity in the spinal cord and brain known as central sensitization. In this state, spontaneous pain could occur and non-noxious mechanical pressures could be magnified to intolerable pain signals (Ji et al., 2018). When it comes to cognitive functions, sustained pain could produce anxiety, which under certain circumstances may result in depression with the pathological sign of dysregulated neurotransmission and cortical reconstruction (Bair et al., 2003; Holmes et al., 2013). In long term, symptoms of muscle spam and deconditioning could also occur, causing functional disability that further decrease the quality of life of patients.

#### 1.3.2 The Dilemma of Single Target Drugs

Classical drug discovery approach has been mainly focused on the screening/designing of compounds selectively against one specific molecular target. These single target drugs (STDs) have been proven to be effective against many types of disease characterized by one predominant alteration and low potential of molecular evolution (Kong WJ. et al., 2020). Few examples of STDs include cyclooxygenase inhibitors that diminish the prostaglandin synthesis for treatment of inflammation and pain, sodium channel blockers for local anesthesia, and beta blockers/angiotension converting enzyme inhibitors for high blood pressure.

However, agents that affect one target only might not always affect complex systems (Csermely et al., 2005). The limitation of STDs can be seen in some challenging clinical situations due to mutation of the target (invading virus, microbes, tumors etc.). For instance, high frequencies of genomic mutations In RNA viruses (such as in HIV or COVID-19) could eventually induce variants that lose the targeted structure, rendering STDs ineffective. Similar scenario applies to many types of tumors (Csermely et al., 2005), under chemotherapy, tumor cells are also subject to high rate of mutations that could enable them to escape from the attacking sites of STDs.

The other confounding factor is alteration of the innate systems (immune system, metabolic system, nervous system etc.), to generate “compensatory” functions that are different enough to not respond to the same drug (Csermely et al., 2005). In chronic diseases (such as metabolic diseases, auto-inflammatory diseases and neurodegenerative diseases), usually there are not only one pathological mechanism that presides the whole duration of the disease. In some cases, the progression of the disease could profoundly modulate the innate systems, to generate robust trends of cellular outputs that unable to be reversed by STDs (Csermely et al., 2005).

For instance, in type II diabetes, STDs could improve high blood sugar/insulin resistance, but not good at controlling the established peripheral damage and associated complications (Tripathi and Srivastava, 2006); Similarly, in rheumatoid arthritis, STDs usually produce poor efficacy on accumulated bone erosion, sensory dysfunctions and comorbidities (Schett and Gravallese, 2012; Coutant and Miossec, 2020); Likewise, in Parkinson’s disease, the pathological mechanism at “subclinical” period is dissimilar to the phase of Parkinsonism when dopaminergic neuronal damage is irreversible by STDs (Gaig and Tolosa, 2009).

All these scenarios demonstrate that in challenging clinical situations (especially in chronic diseases), mutations and pathological and physiological changes across multi-systems/multi-organs, drive effective treatments to cover not only the symptoms, but also the root causes of the disease (Pang et al., 2012).

## 2 Introduction of Traditional Chinese Medicine Derived Analgesic Alkaloids

Facing the insufficiency of therapeutic measures for chronic pain, finding new treatments with stronger analgesic effect but lower side effects, becomes a global mission with ever increasing urgency. In such regard, differed from the STD approach, we turned our interest into traditional Chinese medicine (TCM), which harbors a rich source of drug candidates and usually acting on multiple targets (Zhu et al., 2017; Wang et al., 2019). Among all these potential candidates, many natural alkaloids has been report with analgesic potency (Zhu et al., 2017; Jiang W. et al., 2020). In following paragraph, we will introduce the best-studied analgesic alkaloids derived from TCM.

### 2.1 Tetrahydropalmatine

Tetrahydropalmatine (THP) was originally extracted from the botanic *Corydalis yanhusuo* W.T.Wang [Papaveraceae; Corydalis rhizoma] (Bory et al., 2010), cultivated in the Zhejiang, Jiangxi, and Anhui provinces of China. *Corydalis yanhusuo* W.T.Wang [Papaveraceae; Corydalis rhizoma] was recorded in the first Chinese medical classic, “Shennong Herbal Classic” (compiled in 200 A. D.), listed as a medium-grade drug, and used for invigorating blood circulation, reinforcing vital energy, and relieving pain (Table 1).

**TABLE 1:** Introduction of TCM derived analgesic alkaloids.

Drug	Chemical Structure	Source of origin	Effective pain models	Current clinical application
Tetrahydropalmatine	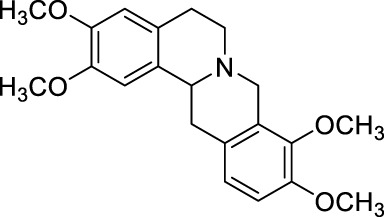	*Corydalis yanhusuo* W.T.Wang [Papaveraceae; Corydalis rhizoma]	Partialsciatic nerve ligation (PSNL) model (Liu et al., 2019) Formalin-induced pain model (Kang et al., 2016) Chronic constriction injury model (Kang et al., 2016) Oxaliplatin-induced neuropathic pain (Guo et al., 2014) Model of bone cancer pain (Zhang M. Y. et al., 2015)	Analgesic, sedative and hypnotic effects on variety of diseases (Ren et al., 2003) Anti-arrhythmia (Ren et al., 2003) Treatment of hypertension (Ren et al., 2003)
Aloperine	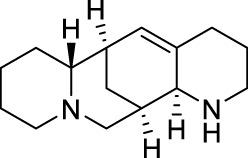	*Sophora alopecuroides* L. [Fabaceae; Sophorae alopecuroides herba]	Chronic constriction injury model (Xu et al., 2014) Acetic acid-induced writhing test (Yang et al., 2015a) Formalin test (Yang et al., 2015a) Mouse ear swelling test (MEST) (Yang et al., 2015a) Carrageenan-induced paw edema test (Yang et al., 2015a)	Treatment of rheumatoid arthritis (Jin et al., 2015; Wang et al., 2020) Treatment of lupus erythematosus (Jin et al., 2015; Wang et al., 2020) Treatment of eczema (Jin et al., 2015; Wang et al., 2020)
Oxysophocarpine	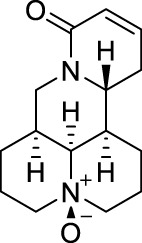	*Sophora alopecuroides* L. [Fabaceae; Sophorae alopecuroides herba]	Mechanical allodynia of the carrageenan-induced paw edema model in mice (Yang et al., 2015b) Tail-flick test (Xu et al., 2013) Hot-plate test (Xu et al., 2013) Acetic acid-induced abdominal constriction (Xu et al., 2013) Formalin-induced pain test (Xu et al., 2013)	NA.
Matrine	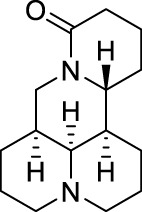	*Sophora flavescens* Ait. [Fabaceae; Sophorae flavescentis radix]	Chronic constriction injury model (Wang eW. t al., 2013) Aceticacid-induced abdominal contraction test (Kamei et al., 1997; Dai et al., 2021) Tail-flick assay (Kamei et al., 1997) Vincristine-induced neuropathic pain model (Dun et al., 2014)	Treatment of atrophic vaginitis (Zhang and Shen, 2021) Treatment of bacterial vaginitis (Zhang and Shen, 2021)
Sinomenine	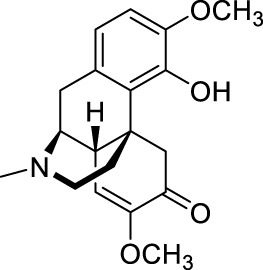	*Sinomenium acutum* (Thunb.) Rehd. et Wils. [Menispermaceae; Sinomenii caulis]	Hot-plate test in rats (Gao et al., 2013; Zhu Q. et al., 2014) Formalin test (Lee et al., 2017) CFA induced inflammatory pain (Li et al., 2017; Yuan et al., 2018) Rhuematoid Arthritic Pain in mice (Gao et al., 2015) Incisional Pain in rats (Zhu et al., 2016; Gao et al., 2021) Cancer-induced bone pain Model (Chen S. P. et al., 2018) Peripheral neuropathic pain in rats and mice (Gao et al., 2013) Central neuropathic pain in rats (Gao et al., 2013; Wang et al., 2021)	Treatment of rheumatoid arthritis (Xu et al., 2008) Treatment of Glomerular disease (Yin et al., 2016) Treatment of systemic lupus erythematosus (Shi et al., 2008; Zeng and Tong, 2020) Detoxification of heroin addicts (Liu Z. et al., 2007)
Ligustrazine	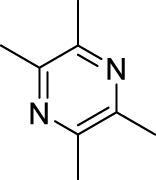	*Ligusticum chuanxiong* Hort. [Apiaceae; Chuanxiong rhizoma]	Carrageenan induced inflammatory pain in mice (Gao et al., 2021) Incisional Pain in rats (Gao et al., 2021) Peripheral Neuropathic Pain in mice (Gao et al., 2019) Central Neuropathic Pain in rats (Gao et al., 2019)	Treatment of ischemic cerebrovascular disease (Li et al., 2009) Treatment of myocardial infarction (Li et al., 2009) Treatment of pulmonary hypertension (Li et al., 2009) Treatment of rheumatoid arthritis (Zhang C. et al., 2019)
Evodiamine	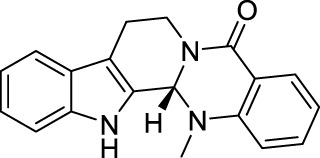	*Tetradium ruticarpum* (A.Juss.) Hart. [Rutaceae; Tetradii ruticarpi fructus]	Hot plate test (Wu and Chen, 2019) Paclitaxel-induced neuropathic pain (Wu and Chen, 2019)	Suppress skin inflammation (Yarosh et al., 2006) Treatment of erythromelalgia (Yarosh et al., 2006) Treatment of Raynaud’s syndrome (Yarosh et al., 2006) Prevention of oral mucosal disorders such as periodontitis, gingivitis or other related oral mucosal inflammation (Zeligs, 2006)
Brucine	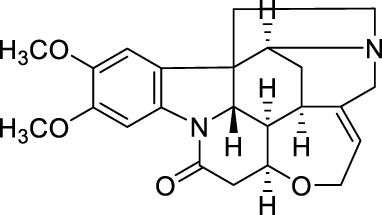	*Strychnos nux-vomica* L. [Loganiaceae; Strychni semen]	Hot-plate test (Yin et al., 2003) Tail-flick test (Yu et al., 2019) Acetic acid-induced writhing test (Yin et al., 2003) Formalin test (Yin et al., 2003) Carrageenan-induced rat paw edema (Yin et al., 2003) Adjuvant-induced arthritis model (Yin et al., 2003) Chronic constriction injury model (Yu et al., 2019)	Treatment of rheumatoid arthritis (Lu et al., 2020) Treatment of traumatic pain (Lu et al., 2020)
Tetrandrine	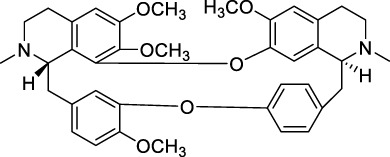	*Stephania tetrandra* S.Moore [Menispermaceae; Stephaniae tetrandrae radix]	Hot-plate test (Zhao et al., 2014) Acetic acid-induced abdominal constriction (writhing) test (Zhao et al., 2014)	Treatment of arthritis (Kong X, et al., 2020) Treatment of arrhythmia (Kong X. et al., 2020) Treatment of hypertension (Kong X. et al., 2020) Treatment of silicosis, pneumoconiosis (Kong X. t al., 2020) Treatment of liver fibrosis (Kong X. et al., 2020) Treatment of portal hypertensive gastropathy (Kong X. et al., 2020)
Stopholidine	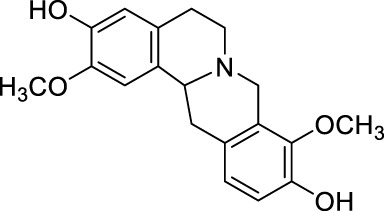	*Stephania japonica* (Thunb.) Miers [Menispermaceae; Stephaniae japonicae folium et rhizome]	Hot-plate test (Chen et al., 1986)	Treatment of schizophrenia (Mo et al., 2007) Treatment of Parkinson’s disease (Yang et al., 2007)
Lappaconitine	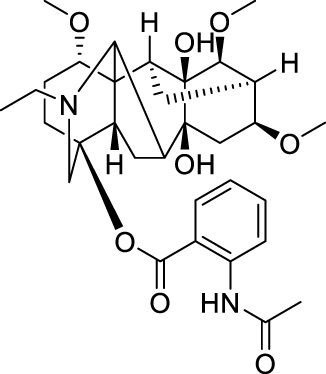	*Aconitum sinomontanum* Nakai [Ranunculaceae; Aconiti sinomontani radix]	Hot-plate test (Ono and Satoh, 1988) Tail immersion test (Ono and Satoh, 1988) Randall-Selitto test (Ono and Satoh, 1988) Acetic acid-induced writhing test (Ono and Satoh, 1988) CCI model of peripheral neuropathic pain (Ou et al., 2011)	Effective for post-operative analgesia with epidural injection (Chen et al., 1996)

As the effective component of *Corydalis yanhusuo* W.T.Wang [Papaveraceae; Corydalis rhizoma], THP also has sedative, hypnotic and especially analgesic effects (Ren et al., 2003) in a variety of pain models, including formalin induced pain (Dosage: 20, 200 nmol) (Kang et al., 2016), bone cancer pain (Dosage: 20, 40, 60 mg/kg) (Zhang MY. et al., 2015) and neuropathic pain induced by sciatic nerve chronic constriction (Dosage: 20 nmol for intraperitoneal injection and 2 nmol for intrathecal injection; Positive control: BD1047 and gabapentin) (Kang et al., 2016) or oxaliplatin (Dosage: 1, 2, 4 mg/kg, Positive control: SCH23390) (Guo et al., 2014). In addition, THP could significantly reverse opioid dependence in rats addicted to morphine (Dosage: 1.25, 2.5, 5 mg/kg; Negative control: morphine) (Jiang WN. et al., 2020). THP has been clinically used in China as analgesics, sedatives, or hypnotics (Ren et al., 2003), and applied for arrhythmia and hypertension treatments (Ren et al., 2003).

### 2.2 Aloperine

Aloperine (Table 1) is a quinolizidine alkaloid isolated from *Sophora alopecuroides* L. [Fabaceae; Sophorae alopecuroides herba] in 1989 (Du and Li, 1989). In TCM, *Sophora alopecuroides* L. [Fabaceae; Sophorae alopecuroides herba] was first mentioned in “Shennong’s Classic of Materia Medica”, and believed to have functions to soothe the five viscera (liver, heart, spleen, lung, and kidney), detoxicate circulation system and stabilize consciousness (Wang et al., 2020). Modern pharmacological studies revealed that aloperine possesses antitumor, antibacterial, antiviral, anti-oxidative, anti-inflammatory, and analgesic properties (Xu et al., 2014; Zhou et al., 2020).

Using experimental animal models, aloperine was found to be effective against inflammatory pain in acetic acid-induced writhing test, formalin test, mouse ear swelling test, carrageenan-induced paw edema test (Dosage: 20, 40, 80 mg/kg; Positive control: aspirin or morphine) (Yang et al., 2015a), and neuropathic pain induced by chronic constriction injury (Dosage: 20, 40, 80 mg/kg; Positive control: pregabalin) (Xu et al., 2014). Clinically, aloperine has been used to treat autoimmune diseases, including rheumatoid arthritis, lupus erythematosus, and eczema, in China (Jin et al., 2015; Wang et al., 2020).

### 2.3 Oxysophocarpine

Similar as aloperine, oxysophocarpine (Table 1) is also extracted from *Sophora alopecuroides* L. [Fabaceae; Sophorae alopecuroides herba] (Du and Li, 1989). Oxysophocarpine has favorable anti-inflammatory and anti-oxidative properties. It could protect neurons going through cell death after oxygen and glucose deprivation (Zhu QL. et al., 2014; Lu et al., 2017; Zhao et al., 2018). Pretreatment with oxysophocarpine could protect against lung injury (Gao et al., 2017).

In addition, oxysophocarpine is an analgesic agent against acute pain in tail-flick or hot-plate test (Dosage: 10, 40, 80 mg/kg; Positive control: morphine) (Xu et al., 2013), inflammatory pain (mechanical allodynia) in carrageenan-induced paw edema model (Dosage: 10, 40, 80 mg/kg; Positive control: aspirin) (Yang et al., 2015b) or formalin-induced pain test (Dosage: 10, 40, 80 mg/kg; Positive control: morphine or aspirin) (Xu et al., 2013), and abdominal pain in acetic acid-induced abdominal constriction model (Dosage: 10, 40, 80 mg/kg; Positive control: morphine or aspirin) (Xu et al., 2013). By so far, we have not found any documentation on well-designed clinical studies of oxysophocarpine yet.

### 2.4 Matrine

Matrine (Table 1) is a quinolizidine alkaloid purified from the root of *Sophora flavescens* Ait. [Fabaceae; Sophorae flavescentis radix] (Lai et al., 2003). The dried root of *Sophora flavescens* Ait. [Fabaceae; Sophorae flavescentis radix] was firstly listed in “Shennong’s Classic of Materia Medica”. The root of *Sophora flavescens* Ait. [Fabaceae; Sophorae flavescentis radix] is bitter in taste, cold in nature and has beneficial properties in relieving rheumatism, improving eyesight, and nourishing the liver and gall (He et al., 2015). Matrine has displayed a broad scope of biological characteristics, including anticancer, anti-inflammatory, anti-allergic, antiviral, antifibrotic, and analgesic effects (Zhang et al., 2020a; You et al., 2020). The spectrum of treatable diseases extends to many systems, such as nervous system, circulatory system, and immune system.

Specifically, matrine could generate antinociceptive effects on variety of pain models, including acute pain in tail-flick test (Dosage: 30 mg/kg; Positive control: pentazocine) (Kamei et al., 1997), abdominal pain in acetic acid-induced abdominal contraction test (Dosage: 1, 3, 10 mg/kg; Positive control: pentazocine) (Kamei et al., 1997; Dai et al., 2021), and neuropathic pain induced by vincristine (Dosage: 15, 30, 60 mg/kg; Positive control: pregabalin) (Dun et al., 2014) or chronic constriction injury (Dosage: 7.5, 15, 30 mg/kg) (Wang et al., 2013a). In China, matrine has been used for clinical conditions of atrophic vaginitis and bacterial vaginitis (Zhang and Shen, 2021), since it can reduce infections, vaginal itching and swelling. Albeit, matrine induces severe side effects, including hepatotoxicity, neurotoxicity, and reproductive and developmental toxicity, thereby limited its clinical usage (You et al., 2020).

### 2.5 Sinomenine

Sinomenine (Table 1) is purified from the root of climbing plant *Sinomenium acutum* (Thunb.) Rehd. et Wils. [Menispermaceae; Sinomenii caulis] (Yamasaki, 1976), which was recorded in the “Compendium of Materia Medica” as an herbal medicine for treatment of various autoimmune diseases, such as rheumatoid arthritis (Zhao et al., 2012). Sinomenine’s pharmacological profile includes immuno-suppression, arthritis amelioration, and protection against hepatitis (Zhao et al., 2012). In addition, evidences revealed sinomenine’s efficacy in alleviating arthritic pain (Xu et al., 2008), and pain in many types of neuralgia, such as sciatic neuritis, lumbalgia and muscular rheumatism (Yamasaki, 1976).

In previous study, we and others had demonstrated that sinomenine possessed a broad spectrum of analgesic effects against acute pain (Dosage: 10, 20, 40 mg/kg) (Gao et al., 2013; Zhu Q. et al., 2014), formalin induced pain (Dosage: 25, 50, 75 mg/kg) (Lee et al., 2017), Freund’s complete adjuvant (CFA) induced inflammatory pain (Dosage: 30, 40 mg/kg) (Li et al., 2017; Yuan et al., 2018), rhuematoid arthritic pain (Model: collagen type II antibody induced arthritis model; Dosage: 10, 20, 40, 80 mg/kg) (Gao et al., 2015), incisional pain (Dosage: 5, 10, 20, 40, 80 mg/kg; Positive control: ligustrazine, paracetamol) (Zhu et al., 2016; Gao et al., 2021), cancer-induced bone pain (Dosage: 10, 20, 40 mg/kg) (Chen SP. et al., 2018), and peripheral (Model: Photochemically induced sciatic nerve injury; Dosage: 10, 20, 40 mg/kg) (Gao et al., 2013) and central neuropathic pain (Model: Photochemically-induced spinal cord injury; Dosage: 10, 20, 40 mg/kg) (Gao et al., 2013). Furthermore, repeated administration of sinomenine did not generate tolerance, but rather increased the basal level of pain threshold (Gao et al., 2014; Gao et al., 2019), suggesting sinomenine’s potential as a long-term therapeutic agent.

Treatment of rheumatoid arthritis is one of the major clinical applications of sinomenine. Compared with nonsteroidal anti-inflammatory drugs, sinomenine was superior in amelioration of morning stiffness, painful joints and erythrocyte sedimentation rate (Xu et al., 2008). In addition, sinomenine is being used for detoxification of heroin addicts (Liu Z. et al., 2007), treatment of patients with systemic lupus erythematosus (Shi et al., 2008; Zeng and Tong, 2020), and decreasing proteinuria and hematuria excretions in patients with glomerular disease (Yin et al., 2016).

### 2.6 Ligustrazine

Ligustrazine (Table 1) is purified from *Ligusticum chuanxiong* Hort. [Apiaceae; Chuanxiong rhizoma] (Li et al., 2011), which is recorded in the “Shennong Herbal Classic” and mainly distributed in Sichuan province (China). The rhizome of *Ligusticum chuanxiong* Hort. [Apiaceae; Chuanxiong rhizoma] is warm in property and pungent in flavor. In a long history, it has been used as folk remedies (for improving blood circulation to protect cardio-cerebrovascular system) and widely applied in food preparation for health promotion (Chen Z. et al., 2018). Ligustrazine has displayed anti-inflammatory, antioxidant, neuroprotective, antifibrotic, antibacterial, and antinociceptive effects (Ozaki, 1992; Jiang and Wang, 2016).

In previous study, we have demonstrated that ligustrazine could effectively reverse carrageenan induced inflammatory pain (Dosage: 10, 20, 80 mg/kg; Positive control: sinomenine, paracetamol) (Gao et al., 2021), incisional pain (Dosage: 10, 20, 80 mg/kg; Positive control: sinomenine, paracetamol) (Gao et al., 2021), peripheral (Model: photochemically induced sciatic nerve injury; Dosage: 10, 20, 80 mg/kg) and central neuropathic pain (Model: photochemically induced spinal cord injury; Dosage: 10, 20, 80 mg/kg) (Gao et al., 2019). By so far, ligustrazine is clinically applied in China for treatment of ischemic cerebrovascular disease (Li et al., 2009), myocardial infarction (Li et al., 2009), pulmonary hypertension and rheumatoid arthritis (Zhang C. et al., 2019).

### 2.7 Evodiamine

Evodiamine (Table 1) is the active component extracted from the fruit of *Tetradium ruticarpum* (A.Juss.) Hart. [Rutaceae; Tetradii ruticarpi fructus] (Moon et al., 1999), which was first recorded in “Shennong’s Classic of Materia Medica”, and has been used as a traditional herbal medicine for more than 2000 years. According to TCM, *Tetradium ruticarpum* (A.Juss.) Hart. [Rutaceae; Tetradii ruticarpi fructus] can disperse cold and relieve pain, arrest vomiting, protect the spleen, help Yang and stop diarrhea (Sun et al., 2020). As the effective component of *Tetradium ruticarpum* (A.Juss.) Hart. [Rutaceae; Tetradii ruticarpi fructus], evodiamine has been reported to exert beneficial effects on atherosclerosis, cardiomyopathy, and ischaemic and valvular heart disease (Li and Wang, 2020). In addition, evodiamine was found to have anti-tumor effects due to its ability in inducing apoptosis, arresting cell cycle, suppressing angiogenesis, and inhibiting tumor migration (Li and Wang, 2020).

In the cell model of transmembrane transportation, evodiamine could easily pass through the blood brain barrier (Sun et al., 2020). Such distribution pattern correlated with evodiamine’s neuroprotective role via regulating neurotrophic factor, and suppressing inflammatory events (Li and Wang, 2020), implying its therapeutic potential for various clinical pain situations. Indeed, researchers have demonstrated evodiamine’s analgesic effect in hot plate test (Dosage: 5 mg/kg) and neuropathic pain model induced by paclitaxel (Dosage: 5 mg/kg) (Wu and Chen, 2019). In clinic, evodiamine is effective in suppressing inflammation in skin and mucosa. It can be used for treating patients with erythromelalgia or Raynaud’s syndrome (Yarosh et al., 2006), and prevention of oral mucosal disorders such as periodontitis, or gingivitis (Zeligs, 2006).

### 2.8 Brucine

Brucine (Table 1), is one of the main bioactive and toxic constituents isolated from *Strychnos nux-vomica* L. [Loganiaceae; Strychni semen] (Tang et al., 2009), which is often used for treating swelling and pain (Guo et al., 2018). Modern pharmacology studies demonstrated brucine’s anticancer effect on many types of tumors, including liver cancer, breast cancer, colon cancer and multiple myeloma (Ma et al., 2013; Lu et al., 2020).

Researchers also validated brucine’s anti-inflammatory and analgesic effects. Brucine was found to induce analgesia in hot-plate test (Dosage: 10.3, 14.7, 20, 30 mg/kg; Positive control: pethidine) (Yin et al., 2003), tail-flick test (Dosage: 10 mg/kg) (Yu et al., 2019), acetic acid-induced writhing test (Dosage: 3.75, 7.5, 15, 30 mg/kg), formalin test (Dosage: 7.5, 15, 30 mg/kg; Positive control: indomethacin), carrageenan-induced inflammatory pain model (Dosage: 15, 30 mg/kg; Positive control: indomethacin), adjuvant-induced arthritis model (Dosage: 15, 30 mg/kg) (Yin et al., 2003), and chronic constriction injury (CCI) model of peripheral neuropathic pain (Dosage: 30 mg/kg; Positive control: gabapentin) (Yu et al., 2019). Clinical applications of brucine are closely related to its analgesic and anti-inflammatory properties. It is typically effective for the treatment of rheumatism, and traumatic pain (Lu et al., 2020). However, brucine showed moderate toxicity on nervous system, immune system, urinary system, and digestive systems (Lu et al., 2020).

### 2.9 Tetrandrine

Tetrandrine (Table 1) is a natural bisbenzylisoquinoline alkaloid purified from the root of *Stephania tetrandra* S. Moore [Menispermaceae; Stephaniae tetrandrae radix] (Chen and Chen, 1935), which was firstly recorded in “Shennong’s Classic of Materia Medica”. *Stephania tetrandra* S. Moore [Menispermaceae; Stephaniae tetrandrae radix] has a diuretic effect and is used in China as a folk remedy for malaria, edema, wet beriberi, dysuria, eczema and inflamed sores (Jiang Y. et al., 2020). As the effective component of *Stephania tetrandra* S. Moore [Menispermaceae; Stephaniae tetrandrae radix], tetrandrine exhibited a variety of therapeutic effects. For instance, anti-tumor properties of tetrandrine had been reported comprehensively based on *in vitro* and *in vivo* studies, against a wide range of cancers (Luan et al., 2020). It has been also found that tetrandrine could exert antimicrobial activity by preventing drug efflux in clinical isolates of treatment-resistant *Mycobacterium tuberculosis* (Zhang Z. et al., 2015). Tetrandrine also displayed antioxidant, anti-inflammatory, anti-allergic, antidiabetic, immunosuppressant, and analgesic effects (Xie et al., 2002; Bhagya and Chandrashekar, 2016).

The antinociceptive effect was validated using animal models of hot-plate test (Dosage: 15, 30, 45 mg/kg; Positive control: morphine) and acetic acid-induced abdominal constriction test (Dosage: 15, 30, 45 mg/kg; Positive control: morphine) (Zhao et al., 2014). Clinically, tetrandrine has been applied to treat arthritis, arrhythmia, hypertension, silicosis, pneumoconiosis, hepatic fibrosis, and portal hypertensive gastropathy (Kong X. et al., 2020). However, the safety, bioavailability, and pharmacokinetic parameters of tetrandrine are still not well studied, especially in clinical settings (Luan et al., 2020).

### 2.10 Stopholidine

Stopholidine (Table 1) is the active component extracted from the botanic *Stephania japonica* (Thunb.) Miers [Menispermaceae; Stephaniae japonicae folium et rhizome] (Brochmann-Hanssen and Richte, 1975), which is traditional used in China for curing asthma, dysmenorrhea, and leprosy (Desgrouas et al., 2014). In the past decades, Chinese researchers have made a great effort to explore the mechanisms of Stopholidine and its potential utility in treating drug abuse, schizophrenia, and pain (Mo et al., 2007; Chu et al., 2008). The evidence of Stopholidine could produce antinociception is primarily revealed by animal model of hot-plate test (Dosage: 20 mg/kg) (Chen et al., 1986). Stopholidine was further shown to have promising potential for clinical use in pain management (Chu et al., 2008). However, the clinical application of stopholidine is still rare and predominantly restricted to treat patients with schizophrenia. Clinical studies revealed that stopholidine has a therapeutic value in schizophrenic patients without produce significant side effects (Mo et al., 2007). Stopholidine also relieved the motor symptoms of Parkinson’s disease when co-administered with Levodopa (Yang et al., 2007).

### 2.11 Lappaconitine

Lappaconitine (Table 1) is a naturally occurring alkaloid purified from the root of *Aconitum sinomontanum* Nakai [Ranunculaceae; Aconiti sinomontani radix] (Bessonova et al., 1990), which by TCM theory possesses a hot and dry nature to resist cold, and being applied as painkillers or antirheumatic agents (Ono and Satoh, 1988; Nyirimigabo et al., 2015). Similarly, lappaconitine is reported to have mixed pharmacological properties, including producing antirheumatic, antiarrhythmic, antiepileptic, and analgesic effects (Nyirimigabo et al., 2015). When examined after oral or subcutaneous administration to rodents in the hot-plate, tail-immersion, or acetic acid-induced writhing tests, lappaconitine exhibited a strong antinociceptive activity, which was even comparable to morphine (Ono and Satoh, 1988). Lappaconitine is also shown to be effective in animal models of acetic acid-induced writhing test (Ono and Satoh, 1988) and CCI model of peripheral neuropathic pain (Dosage: 4 mg/kg) (Ou et al., 2011). In clinical settings, lappaconitine has been successfully used for post-operative analgesia with epidural injection, and generated desired analgesic effects (Chen et al., 1996).

## 3 dCloud Theory

**FIGURE 2:**
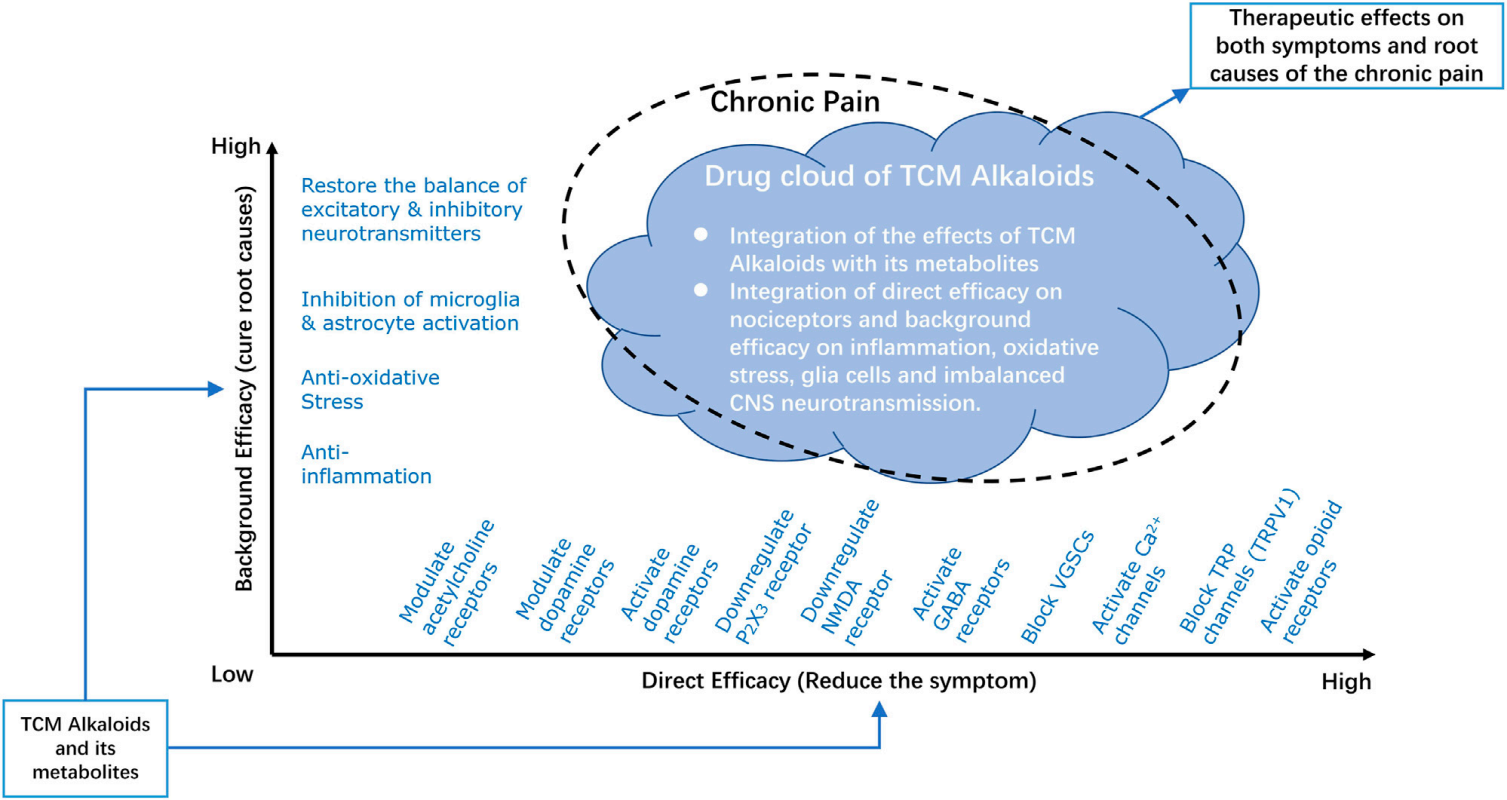
Drug Cloud of TCM derived Analgesic Alkaloids. The integrated analgesic properties of TCM derived alkaloids are illustrated by introducing the notion of drug cloud. The drug cloud could be described from two dimensions, which are direct efficacy and background efficacy. Direct efficacy refers to direct actions of TCM derived alkaloids on nociceptors or pain relaying network through activation/inhibition of ion channels or regulation the expression of certain receptors, such as by downregulating P2X3 or NMDA receptors, activating dopamine, GABA, or opioid receptors, and blocking VGSCs, Ca^2+^, or TRP channels. Background efficacy refers to the ability of TCM derived alkaloids to reverse the root causes of the chronic pain disease, including inhibition of inflammation and oxidative stress, inhibition of microglia and astrocyte activities and restore the balance between excitatory and inhibitory neurotransmitters. The chemical basis of drug cloud is TCM derived alkaloids together with its metabolites. By integration of the direct efficacy and background efficacy, this chemical basis of TCM derived alkaloids could have profound therapeutic effects on both symptoms and root causes of chronic pain. NMDA, N-methyl-d-aspartate; GABA, γ-amino butyric acid; VGSCs, voltage-gated sodium channels; TRP, transient receptor potential; TRPV1, transient receptor potential vanilloid receptor 1.

To better understand the integrated analgesic properties of TCM derived alkaloids, we introduced the notion of “drug cloud” (dCloud) (Kong WJ. et al., 2020), to illustrate their full therapeutic spectrum with two dimensions, which are “direct efficacy” on symptoms and “background efficacy” on the root causes of pain (Figure 2).

### 3.1 Direct Efficacy to Treat Symptoms

Direct efficacy refers to direct actions of TCM derived alkaloids on nociceptors or pain relaying networks in the peripheral nervous system (PNS) and central nervous system (CNS), to suppress pain signal directly (Table 2). Typical direct efficacy can be generated by inhibition of certain ion channels or modulating the expression of receptors that are key to the synaptic transmission.

**TABLE 2:** Mechanism similarity between TCM derived analgesic alkaloids.

Drugs	Symptom/Signs (treatment Targets)	Root Cause Treatment	
		Antioxidant/Anti-Inflammatory Activities	Inhibition of Glial Cell Activation/Restoring the Balance of Neurotransmitters
Tetrahydropalmatine	Activating dopamine D1 and D2 receptors in CNS (Liu et al., 2019); Activating GABAA receptor in CNS (Leung et al., 2003)	Inhibiting proinflammatory mediators such as TNF, IL-1β, and IL-18 in THP-1 cells (Oh et al., 2010); Decreasing the accumulation of inflammatory factors and further suppress oxidative stress in myocardium (Han et al., 2012)	Suppress tumor cell implantation-induced microglial cells activation in CNS (Zhang M. Y. et al., 2015); Reduced the concentrations of Glu and the ratio of Glu/GABA in mice with cerebral ischemia in CNS (Zhang et al., 2004); Reverse the abnormal decrease in dopamine transmitter in morphine-dependent rats in CNS (Jiang W. N. et al., 2020)
Aloperine	No report	Inhibiting NF-κB pathway and downregulating the subsequent expression of proinflammatory cytokines in CNS (Xu et al., 2014); Suppressing the production of ROS while upregulating SOD to suppress oxidative stress in CNS (Xu et al., 2014)	No report
Oxysophocarpine	No report	Suppressing the release of proinflammatory cytokines, such as TNF, IL-1β, IL-6 in PNS (Yang et al., 2015b); Inhibiting the production of nociceptive PGE2 in PNS (Yang et al., 2015b); Activating Nrf2 to regulate an array of detoxifying and antioxidant defense gene expression in lung (Gao et al., 2017)	Increase the expression of GABAAα1 receptors in CNS (Xu et al., 2013)
Matrine	Activating κ-opioid receptors and μ-opioid receptors in CNS (Kamei et al., 1997); Presynaptic activation of acetylcholine receptors in the CNS (Yin and Zhu, 2005)	Inhibiting the release of IL-1 and IL-6 from peritoneal macrophages in mouse peritoneal macrophages (Hu et al., 1996); Suppressing the expression of substance P receptor (NK-1R) in HaCaT cells and fibroblasts (Liu J. Y. et al., 2007); Inhibiting SP-induced IL-1β, IL-8 and MCP-1 production in HaCaT cells and fibroblasts (Liu J. Y. et al., 2007); Attenuating the vincristine-induced inflammation mediated through reduction of myeloperoxidase, TNF, and IL-6 in PNS (Gong et al., 2016); Attenuating the vincristine-induced oxidative stress, manifested by reduction of malondialdehyde, total antioxidant capacity, and total calcium in PNS (Gong et al., 2016)	Exhibit antiepileptic potential by regulating GABA and glutamate levels in the CNS, to promote inhibitory neurotransmission while decreasing glutamate toxicity (Xiang and Jiang, 2013)
Sinomenine	Inhibiting voltage-gated sodium currents in PNS (Lee et al., 2017); Activating GABAA receptors to produce neuroinhibitory effect in CNS (Zhu et al., 2016); Down-regulating the expression of P2X3 receptor in DRG in PNS (Rao et al., 2017); Reducing the excess ATP in neurons and the expression of ATP-gated P2X3 receptors, thereby reducing the activation of NMDA receptors in CNS (Ji et al., 2013; Rao et al., 2017); Activating opioid μ-receptor in CNS (Wang et al., 2008)	Reduction of the expression of various factors related to inflammation, including p38 MAPK, NF-κB, c-fos, p-CAMKII, COX-2, p-CREB, TLR4 and IL-17A in DRG cells *in vitro* (Wang et al., 2021); Inhibiting the transcription factor NF-κB, to further reduce the production of TNF, IL-1β, IL-6, INF-γ, IL-4, and IL-8, thereby alleviates neurogenic inflammation in PNS and in synovial sarcoma (Wang et al., 2005; Li et al., 2006); Inhibiting the expression of pain-inducing PGE2 in CNS (Qian et al., 2007); Upregulating the expression of Nrf2 in CNS (Zhang L. et al., 2019); Upregulating the expression of NADPH in CNS (Qian et al., 2007); Inhibiting the production of ROS, to further prevent the activation of NF-κB and the release of pro-inflammatory cytokines (such as TNF and IL-1β) in CNS (Qian et al., 2007)	Inhibiting the NADPH oxidase in microglial cells in CNS (Qian et al., 2007); Inhibiting the microglial JAK2/STAT3, and the P38 MAPK pathway in CNS (Qian et al., 2007; Chen S. P. et al., 2018; Wang et al., 2021); Downregulating the level of glutamate in extracellular fluid of CNS (Li et al., 2012)
Ligustrazine	Suppressing the expression of P2X3 receptor in primary afferents in PNS (Gao et al., 2008)	Selective suppression of JNK activity, and decrease the expression of MMP-2/9 to generate antinociceptive effect on CCI-induced neuropathic pain in CNS (Jiang et al., 2017); Inhibiting JAK/STAT3 pathway and subsequent down-regulating the elevated levels of TNF-α, IL-1β, and IL-2 in CNS (Wang S. et al., 2016); Inhibiting the edema induced by carrageenin, the increase of the dye leakage induced by acetic acid and the granuloma formation induced by cotton pellet in PNS (Ozaki, 1992); Reducting hydroxyl radicals in hypothalamic regions of the brain in CNS (Chang et al., 2013)	Inhibiting the astrocyte activation in the spinal cord and reduced CCI-induced neuroinflammation in CNS (Jiang et al., 2017); Attenuating activation of microglia and astrocyte in a model of traumatic brain injury in CNS (Wang et al., 2017); Increasing the GABA levels and decreasing the glutamate levels in the CNS to generate neuroprotective activity (Danduga et al., 2018); Reducing hypothalamic levels of glutamate in CNS (Chang et al., 2013)
Evodiamine	Functioning as an agonist for the vanilloid receptor TRPV1 in PNS (Pearce et al., 2004)	Inhibiting inflammatory mediators including IL-1β, IL-6, TNF-α and MCP-1 in PNS (Wu and Chen, 2019); Attenuating adjuvant-induced arthritis in rats by inhibiting synovial inflammation and restoring the Th17/Treg balance in PNS (Zhang H. et al., 2020); Ameliorating inflammatory response through regulating COX-2 and NF-κB signaling pathways in BV-2 cells and in RAW264.7 cells (Liu et al., 2009; Meng et al., 2021); Maintaining mitochondrial anti-oxidant functions and suppressing ROS production in PNS (Wu and Chen, 2019)	Suppressing neuroinflammation caused by over-activated microglias via regulating Akt/Nrf2-HO-1/NF-κB signaling axis *in vitro* (Meng et al., 2021); Reducing caffeine-induced sleep disturbances and excitation in mice via enhancing the expression of γ-aminobutyric acid (GABA)A receptor subunits in the hypothalamus in CNS (Ko et al., 2018)
Brucine	Inhibiting both tetrodotoxin-sensitive (TTXs) and tetrodotoxin-resistant (TTXr) sodium channel in DRG neurons in PNS (Yu et al., 2019)	Inhibiting the release of PGE2 in the inflammatory tissue in CNS and PNS (Yin et al., 2003); Reducting the content of 5-HT in CFA-induced arthritis rat’s blood plasma, while increasing the content of 5-hydroxytryindole-3-acetic acid (5-HIAA) accordingly in CNS and PNS (Yin et al., 2003)	Showing GABA antagonistic property by effectively reversing the inhibitory action of GABA on 35S-TBPS binding *in vitro* (Dalkara et al., 1986)
Tetrandrine	Acting on both L and T-type Ca^2+^ channels to modulates Ca^2+^ mediated signaling events in ventricular cells (Liu et al., 1992)	Inhibiting IKKβ phosphorylation and the COX-2/PGE2 pathway in mice in CNS (Zhao et al., 2014); Inhibiting the production and activation of interleukins, TNF, prostaglandins, cycloxygenase-2 and T cells under experimental conditions to reduce inflammation *in vitro* (Bhagya and Chandrashekar, 2016); Inhibiting freshly fractured quartz-induced lipid peroxidation *in vitro* (Shi et al., 1995); Eliminating hydroxyl radicals *in vitro* (Shi et al., 1995); Scavenging superoxide radicals produced from xanthine/xanthine oxidase *in vitro* (Shi et al., 1995); Reducting hexose-monophosphate shunt activity and hydrogen peroxide production *in vitro* (Seow et al., 1988); Inhibiting the H2O2-induced generation of reactive oxygen species (ROS) in cultured rat cerebellar granule neurons (Koh et al., 2003)	Suppress lipopolysaccharide-induced microglial activation by inhibiting NF-κB pathway *in vitro* (Xue et al., 2008); Suppress LPS-induced astrocyte activation via modulating IKKs-IκBα-NF-κB signaling pathway *in vitro* (Lin et al., 2008); Inhibiting the H2O2-induced elevation of glutamate release in cultured rat cerebellar granule neurons (Koh et al., 2003)
Stepholidine	Partially activating dopamine (DA) D1 receptor while antagonizing D2 receptor activation in the nigrostriatal and mesocorticolimbic DAergic pathways in CNS (Yang et al., 2007; Chu et al., 2008; Ma et al., 2014)	Protecting striatal cells against transient cerebral ischemic injury in CNS (Yang et al., 2007); Scavenging hydroxyl free radicals in CNS (Yang et al., 2007); Maintain neuronal survival following exposure to H2O2 neurotoxicity in CNS (Yang et al., 2007)	No report
Lappaconitine	Use-dependently inhibiting the voltage-gated sodium channels in CNS (Ameri, 1998)	Suppress inflammation *in vitro* (Shaheen et al., 2005); Possess anti-oxidant properties *in vitro* (Shaheen et al., 2005)	No report

#### 3.1.1 Inhibition of Sodium or Transient Receptor Potential Channels

Direct therapeutic efficacy can be achieved through inhibition of ion channels such as blocking voltage gated sodium channels (VGSCs) (Ameri, 1998; Lee et al., 2017; Yu et al., 2019) or transient receptor potential (TRP) channels, especially transient receptor potential vanilloid 1 (TRPV1) (Pearce et al., 2004).

#### 3.1.2 Activation of Dopamine, GABA or Acetylcholine Receptors

Activating the receptors of neurotransmitters such as dopamine (Chu et al., 2008; Liu et al., 2019), or γ-aminobutyric acid (GABA) (Zhu Q. et al., 2014), could also generate direct efficacy against pain. In addition, acetylcholine receptors have emerged as a novel therapeutic target for pain in recent years. Allosteric modulators and silent agonists for acetylcholine receptors have shown to have potential for reducing chronic pain (Toma et al., 2020).

#### 3.1.3 Downregulation of P2X3 and NMDA Receptors

ATP facilitates generation and transmission of the neuropathic pain in dorsal root ganglion (DRG) via the P2X receptors, especially the subtype P2X3. Usually, P2X3 receptor expression is upregulated under neuropathic pain conditions (Ou et al., 2011). Therefore, downregulation of P2X3 receptor may reverse the established trend and generate direct efficacy against chronic pain (Gao et al., 2008; Ou et al., 2011; Rao et al., 2017). Furthermore, downregulating the expression of NMDA receptors could prevent the development of central sensitization, and produce direct antinociceptive effect (Dai et al., 2017).

#### 3.1.4 Activating Opioid μ-Receptor

Receptor phosphorylation is thought to be a pivotal event in agonist regulation of the opioid μ-receptor. The activation/phosphorylation of opioid μ-receptor could be considered as one form of the direct efficacy induced by TCM derived alkaloids (Higashiyama et al., 2005).

### 3.2 Background Efficacy to Treat Root Causes

Background efficacy refers to the ability of TCM derived alkaloids to reverse the root causes of the chronic pain (Table 2), including inhibition of inflammation (Ozaki, 1992; Shaheen et al., 2005) and oxidative stress (Shi et al., 1995; Xu et al., 2014), suppression of microglia/astrocyte activations (Lin et al., 2008; Xue et al., 2008; Zhang MY. et al., 2015), and restoring the balance between excitatory and inhibitory neurotransmission (Xiang and Jiang, 2013; Danduga et al., 2018).

#### 3.2.1 Anti-Inflammation

Inflammation in the nervous system is one root cause of chronic pain. When local inflammation was not well managed, inflammatory mediators (e.g. TNF) released by immune cells (e.g., microglia cells) will sensitize nociceptors (Ji et al., 2016; Jiang W. et al., 2020), and produce persistent pain. In accordance, a number of studies have shown that TNF neutralization antibodies can suppress chronic pain (Marchand et al., 2005; Leung and Cahill, 2010). Thus, anti-inflammation could be a mechanism for background efficacy.

#### 3.2.2 Anti-Oxidation

Oxidative stress emerges when an imbalance exists between free radical formation and the capability of cells to clear them. Under environmental challenge (such as ultraviolet exposure or ischemic damage), the content of free radicals will sharply rise to destroy cellular structures (Pizzino et al., 2017).

Oxidative stress contributes to the establishment and maintenance of chronic pain by exacerbating inflammation and neuropathy (Inoue and Tsuda, 2018; Kaushik et al., 2020). During disease progression, reactive oxygen species (ROS) were accumulated in the CNS, which subsequently activate NMDA receptors, thereby promoting central sensitization (Woolf, 1983). Treatment with ROS scavengers could reverse this effect (Gao et al., 2007), and reduce allodynia induced by nerve damage (Guedes et al., 2008). In addition, superoxide dismutase analogs showed similar effects (reducing inflammation and hyperalgesia) by eliminating ROS (Gao et al., 2007).

#### 3.2.3 Downregulation of Glial Cell Activity

Glial cells (microglial cells and astrocytes), are the most abundant and widely distributed cells which comprises around 90% of the total cell composition in the CNS (Yang and Zhou, 2019). Glial cells are responsible for maintaining a stable environment (clearing the toxic chemicals) and supplying neurons with nutrition. microglial cells serve as the resident macrophages of the spinal cord and brain, spinal microglia have been implicated in the pathogenesis of neuropathic pain after nerve injury (Jessen, 2004; Ji et al., 2016). While astrocytes perform critical functions such as neurotransmitter recycling, formation of the blood-brain barrier, regulation of extracellular ion concentration, and modulation of synaptic transmission. Nerve injury induces myriad changes in astrocytes that lead to enhanced pain (Ji et al., 2016).

Upon activation, glial cells will produce inflammatory factors such as ATP, TNF and interleukin-1β (IL-1β) (Jiang et al., 2020). These inflammatory mediators can dramatically increase the environmental glutamate levels and regulate glutamate transporter in microglial cells (Zumkehr et al., 2018), further enhance the responsiveness of postsynaptic glutamate receptors (such as NMDA and AMPA receptors), and escalate the excitability of the secondary neurons (Yunus, 2015). Glial cell activation eventually results in central sensitization, thereby promoting persistent pain states (Ji et al., 2013; Yunus, 2015). Thereby, enduring beneficial effect could be generated by suppressing the glial cell activity.

#### 3.2.4 Restoring the Homeostasis of Neurotransmitters in Central Nervous System

Excitatory and inhibitory neurotransmitters (including glutamate, dopamine, serotonin, and GABA etc.), play important roles in maintaining the normal function of the CNS and supporting a rich set of functions for cell–cell communication (Hyman, 2005). These neurotransmitters are not only involved in the sensory process but also associated with the affective component of pain (Yam et al., 2018).

Homeostasis between excitatory and inhibitory neurotransmission is dysregulated when chronic pain occurred. For instance, the results of a study using proton magnetic resonance spectroscopy revealed the abnormal increase of glutamate concentration in the CNS in patients with chronic back pain and fibromyalgia (Tzschentke and Schmidt, 2003). On the other hand, in most clinical reports, central GABA concentration in chronic pain patients was not altered (Peek et al., 2020).

As an important root cause of chronic pain, dysregulation of neurotransmitters in the CNS could be detrimental and even result in cortical reconstruction (Elman and Borsook, 2016). Animal study indicated the inhibitory GABA could be converted to a paradoxical excitatory neurotransmitter, following neuronal damage (Hökfelt et al., 1994; Ben-Ari et al., 2012). These reorganizational processes usually produce profound impacts on the sensory system as well as contributed to the establishment of concomitant symptoms such as anxiety and depression (Nutt, 2008). Thus, restoring the homeostasis of neurotransmitters is key to cure the root causes of chronic pain.

### 3.3 Chemical Basis of dCloud

TCM derived alkaloids and its metabolites constituted the chemical basis of drug cloud. By integration of the direct efficacy and background efficacy, chemical basis of TCM derived alkaloids could have profound therapeutic effects on both symptoms and root causes of chronic pain.

## 4 Direct and Background Efficacy of Traditional Chinese Medicine Alkaloids

### 4.1 Tetrahydropalmatine

#### Direct Efficacy

Tetrahydropalmatine (Table 2) generates direct antinociceptive effects via activating the dopamine D1 and D2 receptors (Model: partial sciatic nerve ligation model; Dosage: 10 mg/kg; Positive control: SCH23390, quinpirole) (Liu et al., 2019). However, there are also controversial results indicated that tetrahydropalmatine could block dopamine D2 receptors in the midbrain to regulate nociception (Model: dopamine D2 receptor knockout mice; Dosage: 200 mg/kg; Control: wild type and vehicle) (Wang L. et al., 2016). Further, low dose of tetrahydropalmatine could significantly reduce anxiety in mice, by activation of GABAA receptor (Dosage: 1 mg/kg) (Leung et al., 2003).

#### Background Efficacy

Tetrahydropalmatine (Table 2) inhibits the production of proinflammatory mediators such as TNF, IL-1β and IL-18 (Model: Lipopolysaccharide-stimulated THP-1 cells, bone cancer pain model; Dosage: 0.2, 1, 2 mM, 60 mg/kg; Control: blank, naïve and sham operation) (Oh et al., 2010); Zhang MY. et al., 2015). Besides to decrease the accumulation of inflammatory mediators, it could also reduce the generation of oxidative stress (Model: model of myocardial ischaemia-reperfusion injury; Dosage: 20 mg/kg; Control: vehicle and sham operation) (Han et al., 2012). In addition, tetrahydropalmatine suppressed tumor cell implantation induced activation of microglial cells, which contributed to the attenuation of bone cancer pain (Dosage: 60 mg/kg; Control: vehicle and sham operation) (Zhang MY. et al., 2015). As to restore the balance between excitatory and inhibitory neurotransmission, tetrahydropalmatine markedly reduced the brain concentrations of glutamate, and the ratio of glutamate/GABA in mice with cerebral ischemia (Dosage: 14, 28, 56 mg/kg) (Zhang et al., 2004). Further, the effect of tetrahydropalmatine on opioid dependence, is related to its ability to reverse the abnormal decrease of dopamine (in morphine-dependent rats) and regulate the expression of dopamine receptors (Model: conditioned place preference model; Dosage: 1.25, 2.5 mg/kg) (Jiang WN. et al., 2020).

### 4.2 Aloperine

#### Background Efficacy

Aloperine (Table 2) can inhibit nuclear factor kappa-B (NF-κB) pathway and downregulate the subsequent expression of proinflammatory cytokines (Model: chronic constriction injury model; Dosage: 80 mg/kg; Control: sham operation) (Xu et al., 2014). Aloperine can also reduce the production of ROS while upregulate superoxide dismutase (SOD) to suppress oxidative stress (Model: chronic constriction injury model; Dosage: 80 mg/kg; Control: sham operation) (Xu et al., 2014).

### 4.3 Oxysophocarpine

#### Background Efficacy

Oxysophocarpine (Table 2) exhibited anti-inflammatory effects by suppressing the release of proinflammatory cytokines, such as TNF, IL-1β, IL-6 (Model: carrageenan-induced inflammatory pain model; Dosage: 80 mg/kg; Control: vehicle and sham operation) (Yang et al., 2015b). After the treatment with oxysophocarpine, the xylene-included ear swelling, and carrageenan-induced paw edema decreased as the result of inhibition of prostaglandin E2 (PGE2) (Dosage: 80 mg/kg; Control: vehicle and sham operation) (Yang et al., 2015b). Oxysophocarpine also exerted antioxidant activity by activation of nuclear factor-erythroid 2-related factor 2 (Nrf2), the essential transcription factor that regulates an array of detoxifying and antioxidant defense gene expression (Dosage: 1, 2, μM; Control: blank) (Gao et al., 2017). In addition, oxysophocarpine increased the expression of GABAAα1 receptor in mice to restore the balance between excitatory and inhibitory neurotransmission (Dosage: 80 mg/kg) (Xu et al., 2013).

### 4.4 Matrine

#### Direct Efficacy

Matrine (Table 2) could presynaptically activate acetylcholine receptors in the CNS (Model: hot-plate test; Dosage: 10 mg/kg) (Yin and Zhu, 2005). It could also activate κ-opioid receptors and μ-opioid receptors (Model: acetic acid-induced abdominal constriction test; Dosage: 30 mg/kg) (Kamei et al., 1997) to generate direct efficacy on pain symptoms.

#### Background Efficacy

Matrine (Table 2) exhibited anti-inflammatory properties by inhibiting the production of inflammatory cytokines such as TNF, IL-1β, IL-6 and IL-8 (Model: cultured HaCaT cells and fibroblasts, vincristine-induced neuropathic pain model; Dosage: 10, 50, 100 μg/ml, 60 mg/kg) (Hu et al., 1996; Liu JY. et al., 2007; Gong et al., 2016). Matrine also attenuated the vincristine induced oxidative stress, manifested by reduction of malondialdehyde, total antioxidant capacity, and total calcium (Dosage: 15, 30, 60 mg/kg; Positive control: pregabalin) (Gong et al., 2016). In addition, antiepileptic potential of matrine is realized via regulation of GABA and glutamate levels in the CNS, to promote inhibitory neurotransmission while decreasing glutamate toxicity (Model: maximal electroshock-induced seizures in mice; Dosage: 7.5, 15, 30, 60 mg/kg) (Xiang and Jiang, 2013).

### 4.5 Sinomenine

#### Direct Efficacy

Whole-cell patch clamp recordings of mouse DRG neurons show that sinomenine (Table 2) can dose-dependently reduce voltage-gated sodium currents, thereby reducing pain signal input in the peripheral (Dosage: 3mM, 0.1, 1, 10 mM; Control: blank) (Lee et al., 2017). In addition, the use of GABAA receptor antagonist bicuculine blocked analgesic effect of sinomenine (Zhu Q. et al., 2014), indicating sinomenine’s antinociceptive effect is mediated by the activation of GABAA receptors (Model: chronic constriction injury model; Dosage: 40 mg/kg; Control: vehicle) (Zhu Q. et al., 2014). Besides, sinomenine is able to down-regulate the expression of P2X3 receptor in DRG to suppress diabetic neuropathic pain (Model: type 2 diabetic model rats; Dosage: 40 mg/kg) (Rao et al., 2017). Further, sinomenine has an ability to activate opioid μ-receptor (Model: Chinese hamster ovary cell transfected with opioid μ-receptor; Dosage: 1, 10 μM) (Wang et al., 2008), without generating tolerance (Gao et al., 2014).

#### Background Efficacy

Sinomenine (Table 2) significantly reduced the expression of various factors related to inflammation, including p38 MAPK, NF-κB, c-fos, p-CAMKII, COX-2, p-CREB, TLR4 and IL-17A (Model: cultured dorsal root ganglia cell line, spinal nerve ligation model, cancer-induced bone pain model; Dosage: 800 μmol/L, 20, 40 mg/kg; Control: sham operation and vehicle; Positive control: KN93, SB203580 and oxycodone) (Chen SP. et al., 2018; Wang et al., 2021). Sinomenine can also inhibit the expression of transcription factor NF-κB, to further reduce the production of TNF, IL-1β, IL-6, INF-γ, IL-4, and IL-8, thereby alleviates neurogenic inflammation (Model: collagen-induced arthritis in mice, Freund’s complete adjuvant-induced adjuvant arthritis rats model; Dosage: 30, 100, 300 mg/kg; Positive control: dexamethasone) (Wang Y. et al., 2005; Li et al., 2006; Feng et al., 2007). In addition, sinomenine inhibited the expression of pain-inducing PGE2 (Model: mesencephalic neuron-glia cultures and reconstituted cultures Dosage: 10^−5^ M, 10^−10^ M, 10^−14^ M; Control: blank) (Qian et al., 2007).

As for antioxidant activities, sinomenine may upregulate Nrf2 (Model: spinal cord injury model; Dosage: 40 mg/kg) (Zhang L. et al., 2019) and nicotinamide adenine dinucleotide phosphate oxidase (NADPH) (Model: mesencephalic neuron-glia cultures and reconstituted cultures; Dosage: 10^−5^ M, 10^−10^ M, 10^−14^ M; Control: blank) (Qian et al., 2007), thereby reducing the production of ROS, to play a neuroprotective effect. Besides, by inhibiting the JAK2/STAT3 (Model: cancer-induced bone pain model; Dosage: 40 mg/kg; Control: sham operation and vehicle) (Chen SP. et al., 2018) or P38 MAPK pathway (Model: cultured dorsal root ganglia cell line, spinal nerve ligation model; Dosage: 800 μmol/L, 20 mg/kg; Positive control: KN93, SB203580 and oxycodone) (Wang et al., 2021), sinomenine can suppress the activation of microglia/astrocytes, thereby reducing the production of inflammatory mediators and ATP.

Further, sinomenine has the ability to down-regulate the excessive levels of glutamate in extracellular fluid of CNS, which helps to reconstruct the imbalanced excitatory and inhibitory neurotransmission, in rats with neuropathic pain (Model: spared sciatic nerve injury rat model; Dosage: 20, 40 mg/kg; Positive control: gabapentin) (Li et al., 2012).

### 4.6 Ligustrazine

#### Direct Efficacy

The direct analgesic effect of ligustrazine (Table 2) on neuropathic pain is mediated by suppressing the expression of P2X3 receptor in primary afferents (Model: chronic constriction injury model; Dosage: 100 mg/kg; Control: sham operation) (Gao et al., 2008).

#### Background Efficacy

Ligustrazine (Table 2) inhibited the edema induced by carrageenan, the increase of the dye leakage induced by acetic acid and the granuloma formation induced by cotton pellet (Model: hind-paw edema test, acetic acid-induced vascular permeability test, cotton pellet granuloma inhibition test; Dosage: 30, 100 mg/kg; Positive control: indomethacin) (Ozaki Y, 1992). Ligustrazine could selectively suppress c-Jun N-terminal kinase (JNK) activity, and decrease the expression of MMP-2/9 to generate antinociceptive effect in CCI-induced neuropathic pain model (Dosage: 30 mg/kg) (Jiang et al., 2017). In addition, ligustrazine attenuated neuropathic pain by inhibition of JAK/STAT3 pathway and subsequently reversed the elevated levels of TNF-α, IL-1β, and IL-2 in a rat model of CCI (Dosage: 100 mg/kg; Control: sham operation) (Wang S. et al., 2016).

Ligustrazine can also decrease hydroxyl radicals in hypothalamic regions of the brain to generate antioxidant activities (Model: microdialysis in the hypothalamus of rabbit brain; Dosage: 10, 20, 40 mg/kg) (Chang et al., 2013). Besides, ligustrazine attenuated the activation of microglia/astrocyte in the spinal cord and reduced neuroinflammation (Model: chronic constriction injury model; Dosage: 30 mg/kg) (Jiang et al., 2017; Wang et al., 2017). Further, ligustrazine increased GABA levels while decreasing glutamate levels in the CNS to generate neuroprotective activities and reconstruct the balance between excitatory and inhibitory neurotransmission (Model: 3-NP neurotoxin model; Dosage: 40, 80 mg/kg; Control: blank) (Chang et al., 2013; Danduga et al., 2018).

### 4.7 Evodiamine

#### Direct Efficacy

Evodiamine (Table 2) functions as an agonist for the vanilloid receptor TRPV1, to generate direct efficacy (Model: stably transfected CHO-VR1 cells; Dosage: 0.1–10μM; Positive control: [^3^H] RTX) (Pearce et al., 2004).

#### Background Efficacy

Evodiamine (Table 2) attenuated adjuvant-induced arthritis in rats by inhibiting synovial inflammation and restoring the Th17/Treg balance (Dosage: 10, 20, 40 mg/kg) (Zhang et al., 2020b). Evodiamine also ameliorated paclitaxel-induced neuropathic pain by inhibiting inflammatory mediators including IL-1β, IL-6, TNF-α and MCP-1 (Dosage: 5 mg/kg; Control: vehicle) (Wu and Chen, 2019). Increasing number of studies have demonstrated that evodiamine meliorate inflammatory response through AKT/Nrf2/HO-1 activation and regulating NF-κB signaling pathways (Model: RAW264.7 cells, lipopolysaccharide -stimulated BV-2 cells; Dosage: 0.3, 1, 3, 10, 20 μM; Control: blank) (Liu et al., 2009; Meng et al., 2021).

In addition, evodiamine ameliorates paclitaxel-induced neuropathic pain by maintaining mitochondrial anti-oxidant functions to reduce oxidative stress (Dosage: 5 mg/kg; Control: vehicle) (Wu and Chen, 2019). Evodiamine suppressed over-activated microglias via regulation of environmental inflammatory response (Model: lipopolysaccharide -stimulated BV-2 cells; Dosage: 10, 20μM; Control: blank) (Meng et al., 2021). Further, evodiamine enhanced the expression of GABAA receptor subunits in the hypothalamus and reduced caffeine-induced sleep disturbances (Dosage: 10, 20 mg/kg) (Ko et al., 2018).

### 4.8 Brucine

#### Direct Efficacy

Electrophysiological results show that brucine (Table 2) can directly inhibit the excitability of tetrodotoxin-sensitive and tetrodotoxin-resistant sodium channels in DRG neurons (Model: chronic constriction injury model; Dosage: 30 mg/kg; Positive control: gabapentin) (Yu et al., 2019).

#### Background Efficacy

In formalin test, brucine (Table 2) showed inhibitory effect on carrageenan-induced rat paw edema. It significantly inhibited the release of PGE2 in the inflammatory tissue, reduced acetic acid-induced vascular permeability and the content of 6-keto-PGF1a in blood plasma of CFA induced arthritis rats (Dosage: 15, 30 mg/kg; Positive control: acetylsalicylic acid) (Yin et al., 2003).

In addition, brucine could reduce the content of 5-HT in CFA-induced arthritis rat’s blood plasma, while increase the content of 5-hydroxytryindole-3-acetic acid (5-HIAA) accordingly (Dosage: 15, 30 mg/kg; Positive control: acetylsalicylic acid) (Yin et al., 2003). Besides, *in vitro* study showed that brucine has GABA antagonistic property by effectively reversing the inhibitory action of GABA on 35S-TBPS binding (Model: iontophoretical test on population spikes in the rat hippocampus; Dosage: 0.1 M) (Dalkara et al., 1986).

### 4.9 Tetrandrine

#### Direct Efficacy

Voltage-dependant L-type and T-type calcium channel blockers prevent the movement of Ca^2+^ across the channels, thus controlling signaling events. Tetrandrine (Table 2) acts on both L and T-type Ca^2+^ channels to inhibit Ca^2+^ mediated signaling events in ventricular cells (Liu et al., 1992).

#### Background Efficacy

Tetrandrine (Table 2) is an anti-inflammatory and immunosuppressive agent (Xie et al., 2002). Tetrandrine inhibits the production and activation of interleukins, TNF, prostaglandins, COX-2 and T cells under experimental conditions to reduce inflammation (Bhagya and Chandrashekar, 2016). Antinociceptive effect of tetrandrine on LPS-induced hyperalgesia is mediated by the inhibition of IKKβ phosphorylation and the COX-2/PGE2 pathway in mice (Dosage: 1 × 10^−8^, 1 × 10^−7^, 1 × 10^−6^ mol/L; Control: blank) (Zhao et al., 2014).

In addition, tetrandrine has antioxidant activities. Tetrandrine efficiently eliminated hydroxyl radicals and caused a significant inhibition on freshly fractured quartz-induced lipid peroxidation (Dosage: 60 mM) (Shi et al., 1995). Tetrandrine also scavenged superoxide radicals produced from xanthine/xanthine oxidase (Model: freshly fractured quartz-induced lipid peroxidation; Dosage: 60 mM) (Shi et al., 1995), reduced hexose-monophosphate shunt activity, and inhibited superoxide anion generation (Seow et al., 1988). In addition, tetrandrine inhibited the H2O2-induced generation of ROS in cultured rat cerebellar granule neurons (Dosage: 0.1, 1, 10 μM; Positive control: MK-801, l-NAME, verapamil) (Koh et al., 2003), and exhibited protective effects on hydrogen peroxide-induced oxidative neuronal cell damage in cultured rat cerebellar granule cells (Dosage: 0.1, 1, 10μM; Positive control: MK-801, l-NAME, verapamil) (Koh et al., 2003).

Besides, tetrandrine suppressed lipopolysaccharide-induced microglial activation by inhibiting NF-κB pathway (Dosage: 25, 50 μM; Control: blank) (Xue et al., 2008), and inhibited LPS-induced astrocyte activation via modulating IKKs-IκBα-NF-κB signaling pathway (Dosage: 15, 30 μM; Control: blank) (Lin et al., 2008). Further, tetrandrine could inhibit the H2O2-induced elevation of glutamate release in cultured rat cerebellar granule neurons to restore balance between excitatory and inhibitory neurotransmission (Dosage: 0.1, 1, 10 μM; Positive control: MK-801, l-NAME, verapamil) (Koh et al., 2003).

### 4.10 Stopholidine

#### Direct Efficacy

Stopholidine (Table 2) possesses dopamine D1 partial agonistic and D2 antagonistic properties in the nigrostriatal and mesocorticolimbic DAergic pathways, and can effectively restore the imbalanced functional linkage between D1 and D2 receptors in neurological disorders (Model: heroin-induced reinstatement model; Dosage: 2.5, 5.0, 10.0 mg/kg; Control: Vehicle) (Yang et al., 2007; Chu et al., 2008; Ma et al., 2014).

#### Background Efficacy

Stopholidine (Table 2) exhibits neuroprotective effects through an anti-oxidative mechanism (Yang et al., 2007), in which stopholidine not only protects striatal cells against transient cerebral ischemic injury, but also scavenges hydroxyl free radicals and maintains neuronal survival following exposure to H2O2 induced neurotoxicity (Yang et al., 2007).

### 4.11 Lappaconitine

#### Direct Efficacy

Electrophysiological studies have revealed lappaconitine (Table 2) possesses strong antinociceptive property due to a use-dependent inhibition of the voltage-gated sodium channels (Ameri, 1998). Analgesic effect of lappaconitine also involves the reduction of P2X3 receptor expression and sensitization in rat DRG neurons following CCI (Dosage: 4 mg/kg; Control: sham operation) (Ou et al., 2011).

#### Background Efficacy

There are results indicating lappaconitine (Table 2) exhibited significant anti-inflammatory and anti-oxidant activities (Model: 1,1-diphenyl-2-picryl hydrazyl free radical scavenging activity; Dosage: 100 μg/ml, 1 mM; Control: blank) (Shaheen et al., 2005).

### 5 Drug Combination as a New Therapeutic Approach

The failure of the single target drug discovery model advocates innovation ideas to be adopted in identifying new chronic pain treatment strategies. Natural analgesics introduced in this article is a good resource. But, their potency could be limited, confining their application as mono-therapies. Fortunately, progresses are made in recent years, to demonstrate combinational approach works to improve the clinical efficacy of potential medication (Dale and Stacey, 2016). Empirical evidence shows that 30–50% of the chronic pain patients found beneficial in using drug combinations to control their pain symptoms (Dale and Stacey, 2016).

Therefore, efficient identification of effective combinations could serve as an alternative drug discovery route. In this regard, an ancient Chinese concept could be introduced to treat chronic diseases from a holistic point of view, focusing on promoting the quality of life, instead of completely eliminating the symptom. Simply speaking, the strategy is to combine herbal drugs with different properties (into TCM formulas), to exert effects of mutual reinforcement and detoxification (Chen and Du, 2019). So far, nearly 100,000 formulas have been reported and used in TCM (Zhou and Chen, 2014). Depends on the intrinsic traits of different herbal medicines, TCM formula is a therapeutic regimen containing components of “Jun” (Emperor), “Chen” (Minister), “Zuo” (Assistant), and “Shi” (Delivering Servant) (Zhou and Chen, 2014; Chen, 2016).

“Jun” drug refers to drugs that act on symptom-specific targets in disease-specific tissue (the internal cause) to generate direct efficacy on the major symptom of the disease (in case of chronic pain, blocking ion channels, or directly silencing the signals from pain-relaying neuronal circuits), usually with fast onset but also contribute to majority of the side effects (of the drug combination). “Chen” drug refers to drugs that act on targets against major root causes (both the internal and external causes of disease), to generate primary background effects (in case of chronic pain, suppressing glial cell activation, enhance inhibitory neurotransmission, and to have anti-inflammatory/anti-oxidant effects). The efficacy of “Chen” drug usually accumulates with repeated dosages.

Supplementing “Jun” and “Chen” drugs, “Zuo” drug refers to drugs (usually without toxicity) that act on the external cause of the disease to exhibit salutary and supportive functions, generating desirable protective effects to strengthen the overall health (such as by regulating the composition of gut microbiota or boosting the immune system to combat illness), and in certain cases can mitigate the toxicity (of “Jun” or “Chen” drugs), or improve the compliance (such as by improving flavors of taste). “Shi” drug is the element that acts on absorption/circulation systems (also the external cause of the disease) to facilitate drug (“Jun” or “Chen” drugs) delivery to targeted tissue/organs. Typical “Shi” drug is borneol, which is believed to facilitate the drug penetration across the blood brain barrier to reach CNS (Zhao et al., 2021). In TCM, at least one “Jun” drug is indispensable for the combination therapy to be functional (Wang and Yang, 2013).

**FIGURE 3:**
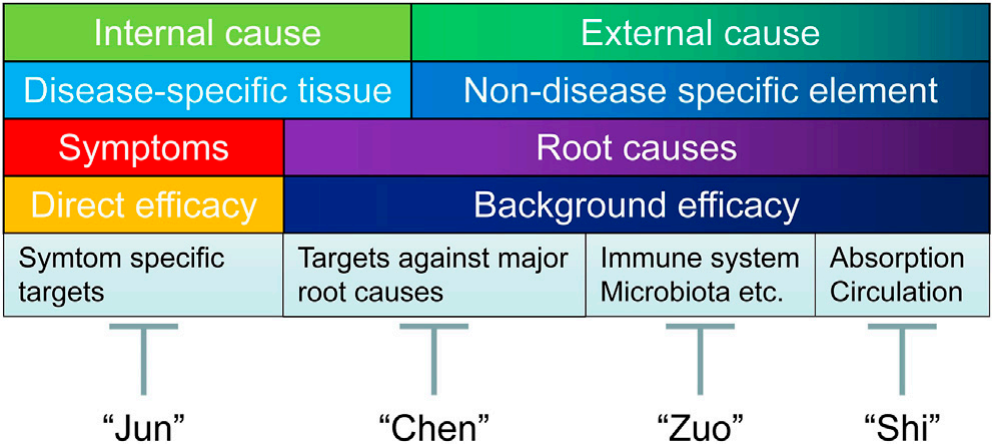
Holistic treatment strategy integrating dCloud and TCM combinational treatment regimen. Above figure illustrated a holistic treatment strategy integrating dCloud and TCM combinational treatment regimen combing herbal drugs with “Jun”, “Chen”, “Zuo”, and “Shi” properties, to exert effects of mutual reinforcement and detoxification. “Jun” drug refers to drugs that act on symptom-specific targets in disease-specific tissue (the internal cause) to generate direct efficacy on the major symptom of the disease (in case of chronic pain, blocking ion channels, or directly silencing the signals from pain-relaying neuronal circuits), usually with fast onset but also contribute to majority of the side effects (of the drug combination). “Chen” drug refers to drugs that act on targets against major root causes (both the internal and external causes of disease), to generate primary background effects (in case of chronic pain, suppressing microglial cell activation, restoring the balance between excitatory and inhibitory neurotransmission, and to have anti-inflammatory/anti-oxidant properties). The efficacy of “Chen” drug usually accumulates with repeated dosages. Supplementing “Jun” and “Chen” drugs, “Zuo” drug refers to drugs acts on the external cause of the disease to have salutary and supportive functions, that often generating desirable protective effects or to strengthen the overall health (such as by regulating the composition of gut microbiota or boosting the immune system to combat illness), and in certain cases can mitigate the toxicity (of “Jun” or “Chen” drugs), or improve the compliance (such as by improving flavors of taste). “Shi” drug is the element that acts on absorption/circulation systems (also the external cause of the disease) to promote drug (“Jun” or “Chen” drugs) delivery to targeted tissue/organs. Typical “Shi " drug is borneol, which is believed to facilitate the drug penetration across the blood brain barrier to reach CNS. In TCM, at least one form of “Jun” drug is indispensable for the combination therapy to be functional.

The theory of “Jun”, “Chen”, “Zuo”, “Shi” is also compatible with the theory dCloud, as illustrated in Figure 3. Combining “Jun”, “Chen”, “Zuo”, and “Shi” drugs, following benefits could be realized (Zhou and Chen, 2014): 1) Increased efficacy on symptoms of chronic disease; 2) Minimized effective dosages; 3) Increased the compliance with mitigation of side effects; 4) Prolonged efficacy over long term usage with less chance of developing tolerance; 5) Restoring the healthy physiological balance to facilitate the overall health.

In light of the above-mentioned concept of “Jun”, “Chen”, “Zuo”, and “Shi”, we interpreted relevant studies, validating drug combinations integrating TCM derived analgesic alkaloids with other components, including natural products, analgesic compounds and other alkaloids (Table 3).

**TABLE 3:** Combinational therapies involving TCM derived analgesic alkaloids.

Types of combination	Drug combinations	Effect
**Alkaloids + Natural Products**	**Tetrahydropalmatine** + *Angelica dahurica* (Fisch, ex Hoffm.) Benth.et Hook.f. [Apiaceae; angelicae dahuricae radix]	*Angelica dahurica* (Fisch, ex Hoffm.) Benth.et Hook.f. [Apiaceae; angelicae dahuricae radix] enhanced the analgesic effects of tetrahydropalmatine in acetic acid writhing test and hot plate test, and improved the plasma concentration of tetrahydropalmatine (Liao et al., 2010)
	**Evodiamine** + Rutaecarpine + Ginsenoside-Rg1+ Ginsenoside-Rb	There is a synergistic interaction between ginsenoside-Rg1, ginsenoside-Rb1, evodiamine and rutaecarpine for effective therapy of mouse migraine (Wu et al., 2016)
	**Sinomenine** + Panax notoginseng saponins	Panax notoginseng saponins did not enhance the analgesic effects of sinomenine in rats with central neuropathic pain (Panax notoginseng saponins alone did not possess analgesic efficacy in this model)
	**Oxymatrine** + sodium ferulate	Oxymatrine combined with sodium ferulate generates significant analgesic effect in acetic acid writhing test and formalin test, which may be related to the synergistic inhibition of transient receptor potential vanilloid-1 (Liu et al., 2010)
**Alkaloids + Analgesic Compounds**	**Matrine** + paracetamol	There is synergistic interaction between matrine and paracetamol in the acetic acid writhing test in mice (Dai et al., 2021)
	**Ligustrazine** + Ketamine	There is an additive interaction between ligustrazine and ketamine in analgesic effect (increased heat radiation-induced tail-flick latency) in normal mice (Liu et al., 2018)
	**Sinomenine** + Paracetamol	Sinomenine combined with paracetamol generated synergistic effect on reducing heat but not mechanical hypersensitivity in carrageenan induced inflammatory pain model in mice (Gao et al., 2021); There is a significant infra-additive interaction between sinomenine and acetaminophen in rat model of incisional pain (Zhu et al., 2016)
	**Sinomenine** + Gabapentin	Sinominine and gabapentin have synergistic effects on peripheral and central neuropathic pain (Gao et al., 2019)
	**Sinominine** + Dextromethorphan	Dextromethorphan did not enhance the analgesic effects of sinomenine in rodents with peripheral or central neuropathic pain (Dextromethorphan alone did not possess analgesic efficacy in these models)
	**Stephenidine** + Dolantin	Systemically applied Stephenidine (2 mg/kg, i.p.) can enhance dolantin (15 mg/kg, i.p.) induced analgesia in hot plate test (in mice) by 175%, to generate synergistic effect (Bian et al., 1985)
**Alkaloids + Alkaloids**	**Sinominine** + Tetrahydropalmatine	There is an additive interaction between sinomenine and tetrahydropalmatine in rat model of central neuropathic pain
	**Sinomenine** + Ligustrazine	Sinomenine facilitates the analgesic efficacy of ligustrazine (in a synergistic way) in rodent models of inflammatory pain, postoperative pain, and peripheral or central neuropathic pain (Gao et al., 2019; Gao et al., 2021)

### 5.1 Tetrahydropalmatine + *Angelica dahurica*

As mentioned, tetrahydropalmatine has direct antinociceptive efficacy by activating dopamine or GABA receptors (Leung et al., 2003; Liu et al., 2019). It also reduced the brain ratio of glutamate/GABA to restore imbalanced neurotransmission (Zhang et al., 2004), and have anti-inflammatory and anti-oxidant activities (Han et al., 2012; Zhang MY. et al., 2015).

On the other hand, *Angelica dahurica* (Fisch, ex Hoffm.) Benth. et Hook. f. [Apiaceae; angelicae dahuricae radix] is known to have natures of activating blood to promote circulation, nourishing liver blood, and driving away cold to relieve pain. Besides, it might increase the plasma concentration of co-applied tetrahydropalmatine (Model: acetic acid-induced writhing test; Dosage: Corydalis alkaloid 17 mg/kg, Angelica dahurica 40 mg/kg, volatile oil 0.05 mg/kg) (Liao et al., 2010).

In this combination, tetrahydropalmatine bares the properties of “Jun” and “Chen” drug (by inducing direct and background effects), while *Angelica dahurica* (Fisch, ex Hoffm.) Benth. et Hook. f. [Apiaceae; angelicae dahuricae radix] could be “Zuo” and “Shi” drug (by strengthen overall health and improve drug exposure). In practice, *Angelica dahurica* (Fisch, ex Hoffm.) Benth. et Hook.f. [Apiaceae; angelicae dahuricae radix] enhanced the analgesic effects of tetrahydropalmatine in acetic acid writhing test and hot plate test, and increased the plasma concentration of tetrahydropalmatine (Liao et al., 2010).

### 5.2 Matrine + Paracetamol

As described above, through modulating opioid or acetylcholine receptors, matrine can generate direct antinociceptive effects (Kamei et al., 1997). It can also produce neuroprotective effects by suppressing the release of inflammatory cytokines (Hu et al., 1996; Liu JY. et al., 2007), reducing the total antioxidant capacity (Gong et al., 2016), or regulating GABA and glutamate levels to diminish the over-excitated neurotransmission (Xiang and Jiang, 2013).

Paracetamol (also known as acetaminophen), could suppress the production of COX dependent PGE2 and preventing the sensitizing of nociceptors (Graham et al., 2013).

In this combination, matrine could be considered as the “Jun” and “Chen” drug, while paracetamol also bares the property of “Chen” drug. In practice, it has been reported that there is a synergistic interaction between matrine and paracetamol in the acetic acid writhing test in mice (Dai et al., 2021).

### 5.3 Oxymatrine + Sodium Ferulate

Similar to matrine, oxymatrine also possess direct antinociceptive effects, and also produces anti-inflammatory and anti-oxidant background effects (Model: synovial tissues gained from RA patients, chronic constrictive injury model; Dosage: 10, 50, 100 μM, 80, 160 mg/kg; Control: blank, sham operation) (Wang et al., 2013b; Liang et al., 2018; Lan et al., 2020).

On the other hand, sodium ferulate induces vasodilation, promotes blood circulation, and has anti-thrombosis, anti-inflammatory, and anti-oxidant effects (Model: xylene-induced mouse ear edema model, carrageenan-induced rat paw edema model, lipopolysaccharide-activated RAW 264.7 cells; Dosage: 7.7, 15.4, 30.9 mg/kg, 50, 100, 200, 400 μmol/L; Positive control: dexamethasone) (Wang and Ou-Yang, 2005; Yuan et al., 2011). In addition, sodium ferulate is also frequently used for strengthen the body’s immunity (Hu et al., 2017).

In this combination, oxymatrine could be considered as the “Jun” drug, while sodium ferulate is suitable to be both “Chen” and “Zuo” drug. In practice, oxymatrine combined with sodium ferulate produced synergistic analgesic effect in acetic acid writhing test and formalin test (Dosage for sodium ferulate + oxymatrine: 24.5 + 49.0, 49.0 + 98.0, 98.0 + 196.0 μmol/kg; Positive control: diclofenac) (Liu et al., 2010).

### 5.4 Evodiamine + Rutaecarpine + Ginsenoside-Rg1 + Ginsenoside-Rb

Evodiamine activates TRPV1 to produce direct antinociceptive effects (Pearce et al., 2004). It also inhibits the release of inflammatory cytokines and ROS to reduce oxidative stress (Wu and Chen, 2019). In addition, evodiamine could suppress neuroinflammation caused by over-activated microglias (Meng et al., 2021), and restore the imbalanced neurotransmission via enhancing the expression of GABAA receptor subunits in the CNS (Ko et al., 2018).

Rutaecarpine is believed that have property of dispelling cold and relieving pain. Evodipine can inhibit the expression of COX-2 and inducible nitric oxide synthase (iNOS) in a dose-dependent manner, further reducing the release of PGE2 (Liu et al., 2009).

Ginsenosides are a set of steroid compounds, mainly exist in ginseng genus of medicinal botanics. Ginsenosides can increase the vitality and quantity of NK cells, B cells, and bone marrow cells. At the same time, ginsenosides can also increase the concentration of immune mediators such as IL-2 and IFN-γ (Lee et al., 2004; Lv et al., 2014). Ginsenoside-Rg1 and ginsenoside-Rb, could boost the immune system and strengthen the overall health, protecting the host from cancer cell invasion and metastasis (Model: murine macrophage-like RAW264.7 cells and human THP-1 monocyte; Dosage: 5, 10, 20, 40, 60, 80, 100, 120 μM; Control: blank) (Li et al., 2018; Ren et al., 2020). They can also promote the resolution of inflammation, and accelerate wound recovery (Model: spinal cord injury model; Dosage: 10 mg/kg; Control: vehicle) (Ahmed et al., 2016; Gao et al., 2020; Xu et al., 2020).

In this combination, evodiamine is both the “Jun” and “Chen” drug, while, rutaecarpine is “Chen” drug, and ginsenoside-Rg1 with ginsenoside-Rb are “Zuo” drugs. In practice, it has been demonstrated that there is a synergistic interaction between evodiamine, rutaecarpine, ginsenoside-Rg1, and ginsenoside-Rb1 for the treatment of migraine (Wu et al., 2016).

### 5.5 Ligustrazine + Ketamine

As mentioned, ligustrazine suppresses the expression of P2X3 receptor in primary afferents to directly modulate nociception (Gao et al., 2008). It also exhibited anti-inflammatory and anti-oxidant properties, and attenuate activation of microglia and astrocytes (Chang et al., 2013; Wang S. et al., 2016; Jiang et al., 2017). In addition, ligustrazine increased GABA levels while decreased glutamate levels in the CNS to generate neuroprotective activity (Danduga et al., 2018).

Ketamine is used to treat various chronic pain syndromes, especially those that have a neuropathic component (Niesters et al., 2014). Low dose ketamine produces strong analgesia in neuropathic pain states, presumably by inhibition of the NMDA receptor, though other mechanisms are possibly involved, including enhancement of descending inhibition (Niesters et al., 2014).

In this combination, Ketamine is the “Jun” drug, while ligustrazine is the both the “Jun” and “Chen” drug. In practice, there is an additive interaction between ligustrazine and ketamine in analgesic effect against acute pain (Model: heat radiation-induced tail-flick test) in normal mice (Liu et al., 2018).

### 5.6 Sinomenine + Ligustrazine

As stated, sinomenine is an analgesic alkaloid that can act on both the peripheral and the central nervous system. It can generate direct analgesic effect by blocking ion channels to stop the firing of pain-relaying neurons (Lee et al., 2017), as well as activating GABAA receptor or opioid μ-receptor (Wang et al., 2008; Zhu et al., 2016), and down-regulating the expression of P2X3 receptor in DRG (Rao et al., 2017). Besides, sinomeine also has profound anti-inflammatory and anti-oxidant activities, and it inhibits activation of microglial cells (Qian et al., 2007; Wang et al., 2021). In addition, sinomenine might mitigate the glutamate toxicity in neuropathic states, by reducing the excessive levels of glutamate in CNS (Li et al., 2012).

In this combination, sinomenine together with ligustrazine are both “Jun” and “Chen” drugs. Ligustrazine failed to perform as a “Shi” drug for sinomenine since co-applied ligustrazine (i.v.) could not enhance sinomenine’s penetration through BBB into the brain (Gao et al., 2021). In practice, sinomenine facilitated the analgesic efficacy of ligustrazine (in a synergistic way) on inflammatory pain, postoperative pain (Gao et al., 2021), and peripheral or central neuropathic pain in rodent models (Gao et al., 2019).

### 5.7 Sinomenine + Paracetamol

In this combination, sinomenine is both the “Jun” and “Chen” drug, while paracetamol has the property of “Chen” drug. In practice, the overall efficacy of this combination varies between pain models and pain modalities. Sinomenine combined with paracetamol generated synergistic effect in reducing heat but not mechanical hypersensitivity in carrageenan induced inflammatory pain model in mice (Gao et al., 2021). And surprisingly, there is a significant infra-additive interaction between sinomenine and acetaminophen in rat model of incisional pain, which could be related to pharmacokinetic interaction between the two drugs (Zhu et al., 2016; Gao et al., 2021).

### 5.8 Sinomenine + Gabapentin

Gabapentin was firstly developed as an antiepileptic drug, increasing the efficacy of the inhibitory neurotransmitter GABA. However, its principal analgesic mechanism is through modulating the activities of the alpha 2-delta subunit of L-type voltage-regulated calcium channels rather than solely enhancing GABAergic neurotransmission (Striano and Striano, 2008).

In this combination, gabapentin is the “Jun” drug, while sinomenine functions as the “Jun” and “Chen” drug. In practice, sinominine plus gabapentin generated synergistic effects on peripheral and central neuropathic pain in animal models (Gao et al., 2019).

### 5.9 Sinominine + Tetrahydropalmatine

In this combination, sinomenine together with tetrahydropalmatine both function as the “Jun” and “Chen” drugs. In practice, we found an additive interaction between sinomenine and tetrahydropalmatine in rat model of central neuropathic pain (Unpublished data).

### 5.10 Sinominine + Dextromethorphan

Dextromethorphan is a weak NMDA antagonist (Stahl, 2019) and commonly used as antitussive agent (Nguyen et al., 2016). It has beneficial effects across a variety of neurological disorders including traumatic brain injury, seizure, pain, methotrexate neurotoxicity, Parkinson’s disease and autism (Nguyen et al., 2016).

In this combination, sinomenine is both the “Jun” and “Chen” drug, while dextromethorphan is considered as the “Chen” drug. In practice, surprisingly, we found dextromethorphan did not enhance the analgesic effects of sinomenine in rodents with peripheral or central neuropathic pain. In addition, dextromethorphan alone did not possess analgesic efficacy in these models (Unpublished data).

### 5.11 Sinomenine + Panax notoginseng Saponins

Panax notoginseng saponins are the main ingredients of *Panax notoginseng* (Burk) F. H. Chen [Araliaceae; notoginseng radix et rhizoma], being classified into four types: protopanaxadiol, protopanaxatriol, ocotilloltype, and oleanolic acid constituents. Panax notoginseng saponins are also the main effective constituents of Xuesaitong Injection, which is widely used in the treatment of cerebral ischemic stroke and cardiovascular disease in China (Zhang X. et al., 2015). Panax notoginseng saponins have neuroprotective and anti-oxidant activities via reducing the levels of ROS, malondialdehyde and nitric oxide (Model: cisplatin-induced nephrotoxicity model; Dosage: 31.35 mg/kg; Control: vehicle) (Li et al., 2020a), while increasing the levels of superoxide dismutase, catalase and glutathione (Qu et al., 2020). Panax notoginseng saponins are also found to be able to modulate gut microbiota and boost the immune system to strengthen the overall health (Model: lincomycin hydrochloride-induced microbial intestinal disorder model; Dosage: 20, 60 mg/kg) (Sun et al., 2003; Song and Hu, 2009; Bai et al., 2021).

In this combination, sinomenine is both the “Jun” and “Chen” drug, while panax notoginseng saponins is both the “Chen” and “Zuo” drug. In practice, panax notoginseng saponins did not enhance the analgesic effects of sinomenine in rats with central neuropathic pain. In addition, panax notoginseng saponins alone did not possess analgesic efficacy in this model (Unpublished data).

### 5.12 Stopholidine + Dolantin

As mentioned, stopholidine could modulate the activity of dopamine receptors which may contribute to its direct effects on nociception (Yang et al., 2007; Chu et al., 2008; Ma et al., 2014). In addition, Stopholidine exhibits neuroprotective effects through an anti-oxidative mechanism (Yang et al., 2007).

Dolantin was developed as an anti-spasmodic analgesic in 1939 (Ngan Kee, 1998). It is the first synthetic opioid, which mainly activating the opioid μ receptors, with a rapid onset and short duration of action (Thigpen et al., 2019). Clinically, dolantin is used for moderate to severe pain relief, such as for post-traumatic pain. Even evidences has challenged dolantin’s analgesic benefits (with concerns have been raised about its safety profile), the drug remains frequently used (Ching Wong and Cheung, 2020).

In this combination, dolantin is the “Jun” drug, while Stopholidine might be the “Jun” and “Chen” drug. In practice, systemically applied Stephenidine (2 mg/kg, i. p.) can enhance dolantin (15 mg/kg, i. p.) induced analgesia in hot plate test (in mice) by 175%, to generate synergistic effect (Bian et al., 1985).

## 6 Conclusion

The dilemma for drug development in chronic pain filed represents the typical failure of the current drug discovery mode, which mainly focused on validating one drug target, and designing molecules solely act on that target (Csermely et al., 2005). Differed from classical STDs, TCM analgesic alkaloids have different pattern of action. Specifically, through pleiotropic activities on multiple targets and pathways essential to pathogenesis and progression of chronic pain, they could provide treatments both on symptoms/signs and root causes. As natural compounds, TCM analgesic alkaloids usually possess moderate potency, but their long-term adverse effects are lower especially compared with opioids (Du et al., 2016), thus are potential drug candidates.

In TCM, normally drugs are combinedly used to boost the overall efficacy (Chen and Du, 2019). The same philosophy could be applied in the identification of effective therapeutic combinations between TCM analgesic alkaloids and other established treatments. In this article, we provided examples on how TCM analgesic alkaloids could enhance the therapeutic potency of other drugs on chronic pain, provided a basis for future investigation to identify and evaluate the most promising combinations.

As the booming in the application of computer and artificial intelligence (AI) technologies, traditional industries are being transformed. We are facing an era that our existing mode of the drug discovery to be modulated by AI based technologies (such as deep learning algorithm), especially in the drug designing process (Hessler and Baringhaus, 2018). For instance, new technologies might enable the chemists to deliberate a drug design that rationally assembles a so called “fusion compound” by assembling functional groups associated with both symptoms/signs and root causes. Integrating these approaches with comprehensive pharmaceutical and pharmacological studies may revolutionize the current mode of drug discovery.

We see in recent years, the number of approved TCM drugs dropped rapidly in China. We believe that this is related to the current evaluation standard of the Chinese authority (Center for Drug Evaluation), solely focusing on the short-term improvement on symptoms. While TCM often has low toxicity, and by targeting the root causes of the disease, bring beneficial outcome in long-term including less adverse events and a higher quality of life, especially for chronic diseases. The current situation call for the establishment of innovative standards for evaluating TCM drugs. Such as by setting up new criterion for long-term safety testing and patient’s quality of life assessment. This is also the area that future studies could help to provide scientific evidence in framing these protocols and eventually facilitate clinical translation.
